# New Sesquiterpene Glycosides from the Flowers of *Aster koraiensis* and Their Inhibition Activities on EGF- and TPA-Induced Cell Transformation

**DOI:** 10.3390/plants12081726

**Published:** 2023-04-20

**Authors:** Young-Hye Seo, Ji-Young Kim, Seung-Mok Ryu, Sun-Young Hwang, Mee-Hyun Lee, Nahyun Kim, Hojun Son, A-Yeong Lee, Hyo-Seon Kim, Byeong-Cheol Moon, Dae-Sik Jang, Jun Lee

**Affiliations:** 1 Herbal Medicine Resources Research Center, Korea Institute of Oriental Medicine (KIOM), Naju 58245, Republic of Korea; 2 Department of Biomedical and Pharmaceutical Sciences, Graduate School, Kyung Hee University, Seoul 02447, Republic of Korea; 3 College of Korean Medicine, Dongshin University, Naju 58245, Republic of Korea; 4 Division of Forest Industrial Materials, Department of Forest Products and Industry, National Institute of Forest Science, Seoul 02455, Republic of Korea; 5 Forest Medicinal Resources Research Center, National Institute of Forest Science, Yeongju 36040, Republic of Korea

**Keywords:** *Aster koraiensis*, sesquiterpene glycoside, anticancer, epidermal growth factor, 12-*O*-tetradecanoylphorbol 13-acetate, cell transformation

## Abstract

In total, four new eudesmane-type sesquiterpene glycosides, askoseosides A–D (**1**–**4**), and 18 known compounds (**5**–**22**) were isolated from the flowers of *Aster koraiensis* via chromatographic techniques. Chemical structures of the isolated compounds were identified by spectroscopic/spectrometric methods, including NMR and HRESIMS, and the absolute configuration of the new compounds (**1** and **2**) was performed by electronic circular dichroism (ECD) studies. Further, the anticancer activities of the isolated compounds (**1**–**22**) were evaluated using the epidermal growth factor (EGF)-induced as well as the 12-*O*-tetradecanoylphorbol 13-acetate (TPA)-induced cell transformation assay. Among the 22 compounds, compounds **4**, **9**, **11**, **13**–**15**, **17**, **18**, and **22** significantly inhibited both EGF- and TPA-induced colony growth. In particular, askoseoside D (**4**, EGF: 57.8%; TPA: 67.1%), apigenin (**9**, EGF: 88.6%; TPA: 80.2%), apigenin-7-*O*-*β*-d-glucuronopyranoside (**14**, EGF: 79.2%; TPA: 70.7%), and 1-(3′,4′-dihydroxycinnamoyl) cyclopentane-2,3-diol (**22**, EGF: 60.0%; TPA: 72.1%) showed higher potent activities.

## 1. Introduction

*Aster koraiensis* Nakai (syn. *Asteromoea koraiensis* and *Gymnaster koraiensis*), belonging to the family Compositae (Asteraceae), is an endemic plant in the Republic of Korea distributed throughout the Korean peninsula. The plant presents beautiful pale purple flowers, which are used as tea and the young shoots are used as a food ingredient. Traditionally, extracts of the aerial parts and roots of *A. koraiensis* are used to treat pertussis, pneumonia, and chronic bronchitis [1,2]. Further, the extracts have also been reported to be anti-diabetic [3,4], antinociceptive [5], liver protective [6], and anti-inflammatory [7] activities. Several phytochemical studies have reported the presence and biological effects of sesquiterpenoids, polyacetylenes, flavonoids, caffeoylquinic acids, and saponins in *A. koraiensis* [2,8,9,10]. Recently, it has been reported that astersaponin I, saponin isolated from the aerial parts of this plant, inhibits the SARS-CoV-2 infection [11]. Most of these studies have been conducted on the aerial parts, roots, and leaves of this plant, and, currently, research on components and physiological activities specific to the flowers of this plant is relatively lacking compared to that of other parts of the plant. In the case of Compositae plants, since the flower parts are often used for edible or medicinal purposes, many studies on the components and efficacy specific to the flower part have been conducted [12,13,14,15,16,17,18,19], but studies on the flower of this plant have not been sufficiently conducted. Therefore, it is necessary to study the components of the flower of this herb and the efficacy of these components.

Cancer is a major cause of death in humans, worldwide, and approximately one in five people develop cancer during their lifetime [20]. As a result of continuous research over the past decades to discover new anticancer compounds from various natural products, such as plants, and to identify their anticancer properties, about 25% of all anticancer drugs approved in 1981 and 2019 were related to natural products. As such, natural products are a rich source of numerous bioactive compounds that have potential as therapeutic agents for various diseases, including cancer [21]. In addition, natural compounds are less toxic and effective in cancer prevention and treatment [22].

As part of the research to discover new bioactive substances of plants distributed in Korea, the flower extract of *A. koraiensis*, which showed a significant anti-carcinogenic effect through our preliminary screening, was selected as the target plant for this study. As mentioned above, this plant is endemic to Korea, but little is known about its cancer-preventive constituents, especially in its flowers. The anti-carcinogenic activity in this study was evaluated by the epidermal growth factor (EGF)-induced and 12-*O*-tetradecanoylphorbol 13-acetate (TPA)-induced cell transformation assays. Neoplastic cell transformation is one of the basic mechanisms of tumorigenesis, and various cell transformations can be induced by carcinogens, such as EGF and TPA [23]. EGF signaling is known to stimulate cell proliferation and survival in several types of human cancer cells [24,25,26]. In addition, treatment with TPA, a skin tumor promoter, induces transformation and forms colonies in soft agar [27]. Therefore, substances that inhibit cell transformation by these carcinogens, such as EGF and TPA, can be considered to have anti-carcinogenic efficacy.

In this study, four new eudesmane-type sesquiterpenes (**1**−**4**), along with 18 compounds (**5**−**22**) were isolated from the flowers of *A. koraiensis* via chromatographic separation methods and their anti-carcinogenic activities were evaluated by EGF- and TPA-induced cell transformation assay.

## 2. Results and Discussion

### 2.1. Structural Elucidation of Compounds

**Figure 1:**
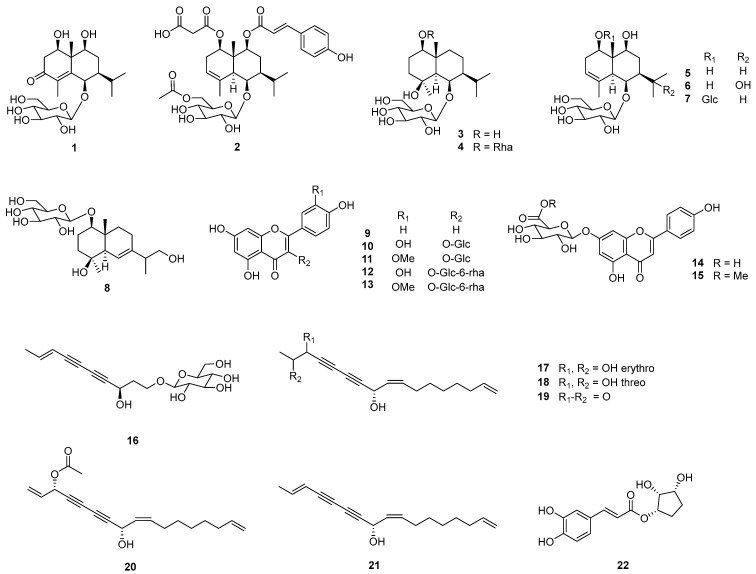
Structures of isolated compounds from the flowers of *Aster koraiensis*.

Four new eudesmane-type sesquiterpenes (**1**−**4**), along with 18 known compounds (**5**−**22**) were isolated from the flowers of *A. koraiensis*, and identified through spectroscopic/spectrometric methods, such as NMR, HRESIMS, and ECD (Figure 1).

**Figure 2:**
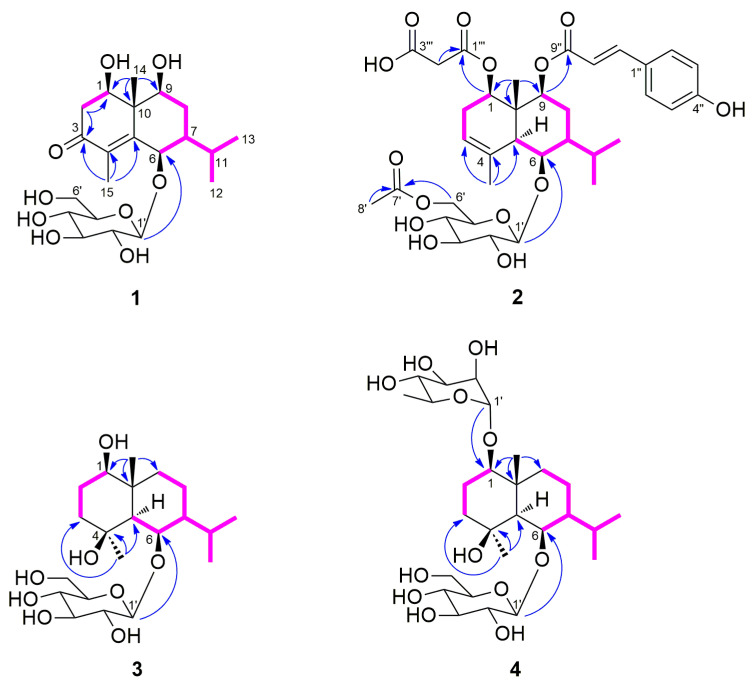
Key COSY (▬) and HMBC (→) correlations of compounds **1−4**.

**Figure 3:**
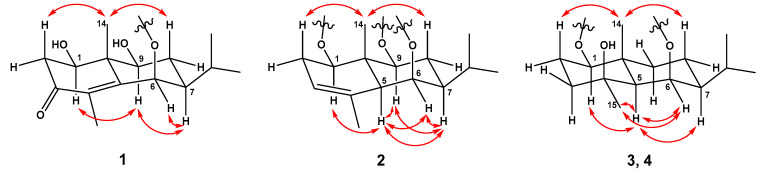
Key NOESY(↔) correlations of compounds **1−4**.

**Figure 4:**
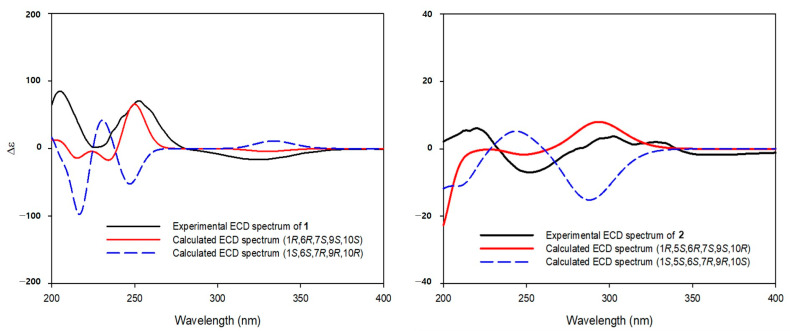
ECD spectra of compounds **1** and **2**.

The molecular formula (C_21_H_34_O_9_) of compound **1** was identified by the HRESIMS. The ^1^H NMR spectrum showed signals of four methyl groups, [*δ*_H_ 0.99 (H-12), 1.03 (H-13), 1.36 (H-14), and 1.85 (H-15)]; two methylene groups, [*δ*_H_ 1.78 (H-8*α*), 1.86 (H-8*β*), 2.45 (H-2*α*), and 2.66 (H-2*β*)]; three oxygenated methine protons, [*δ*_H_ 3.65 (H-9), 3.99 (H-1), and 4.89 (H-6)]; two methine protons, [*δ*_H_ 1.04 (H-7) and 2.03 (H-11)]; and a *β*-glucopyranosyl unit containing an anomeric proton, (*δ*_H_ 4.37, H-1′). The ^13^C NMR and HSQC spectra displayed signals for 21 carbons, including a carbonyl group, [*δ*_C_ 200.8 (C-3)]; two unprotonated sp^2^ carbons, [*δ*_C_ 134.1 (C-4) and 159.0 (C-5)]; and a quaternary carbon, [*δ*_C_ 46.6 (C-10)] (Table 1). The 1D NMR data showed that compound **1** is a glycosylated eudesmane-type sesquiterpene. The positions of three hydroxyl and isopropyl groups were assigned using COSY correlation peaks to be C-1, C-6, C-9, and C-7, respectively (Figure 2). In the HMBC spectrum, the carbonyl group position was determined as a C-3 position by cross peaks of H-15 with C-3/C-4/C-5 and H-2 with C-1/C-3/C-4/C-10. The linkage between glucopyranose and aglycone was also confirmed from the HMBC correlation peak of H-1′ with C-6. The relative configuration of compound **1** was confirmed from the NOESY spectrum (Figure 3). The correlation peaks of H-1 with H-9, H-9 with H-7, and H-7 with H-6 indicated that C-1, C-6, C-7, and C-9 possess the same configurations, whereas H-14 correlated only with H-2*β*/H-8*β.* These data indicated that the relative configurations of C-1, C-6, C-7, C-9, and C-10 positions are all in the β-form (HRESIMS and NMR spectra of 1: Supplementary Figures S1–S7). The (1*R*,6*R*,7*S*,9*S*,10*S*)-absolute configuration of **1** was identified by the calculated and experimental ECD spectra (Figure 4). The sugar units of compounds **1**–**4** were identified as l-rhamnopyranose and d-glucopyranose moieties through UPLC analysis of the reactants following hydrolysis by comparing their retention times to those of standard derivatives. Thus, compound **1** (askoseoside A) was established as (1*R*,6*R*,7*S*,9S,10*S*)-1,6,9-trihydroxy-eudesm-4-en-3-one-6-*O*-*β*-d-glucopyranoside.

**Figure 5:**
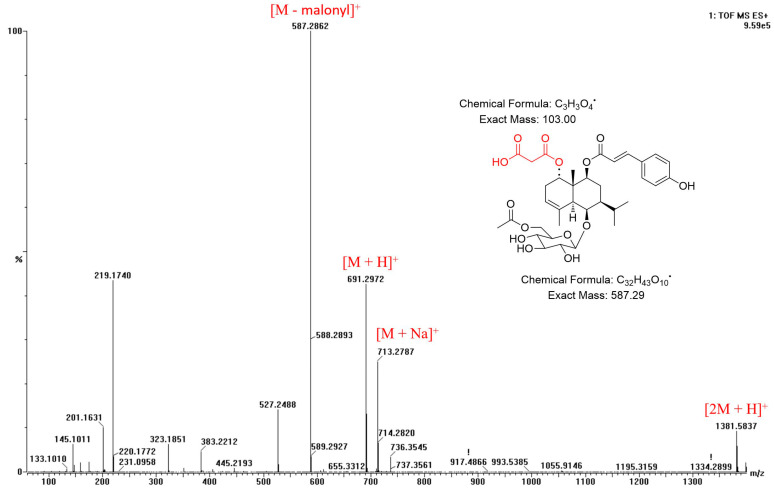
HRESIMS fragment data (positive) of compound **2**.

The molecular formula of compound **2**, C_35_H_46_O_14_, was determined by the HRESIMS data. The ^1^H NMR data of compound **2** displayed the signals of five methyl groups, [*δ*_H_ 0.92 (H-13), 1.02 (H-12), 1.36 (H-14), 1.83 (H-15), and 2.05 (H-8′)]; three methine protons, [*δ*_H_ 1.17 (H-7), 2.09 (H-5), and 5.26 (H-3)]; a *trans*-olefinic group, [*δ*_H_ 6.32 (H-8″) and 7.61 (H-7″)]; *p*-disubstituted aromatic protons, [*δ*_H_ 6.80 (H-3″, 5″) and 7.47 (H-2″, 6″)]; and a *β*-glucopyranosyl unit. In the ^13^C NMR spectrum (Table 1), signals for two carbonyl carbon, [*δ*_C_ 168.7 (C-9″) and 172.7 (C-7′)]; three unprotonated sp^2^ carbons, [*δ*_C_ 135.4 (C-4), 127.3 (C-1″), and 161.3 (C-4″)]; and a quaternary carbon, [*δ*_C_ 42.2 (C-10)], were observed. The 1D NMR spectra of compound **2** were similar to those of reported analogue, (1*R*,5*S*,6*R*,7*S*,9*S*,10*R*)-9-*O*-(*E*-*p*-coumaroyl)-1,6,9-trihydroxy-eudesm-3-en-6-*O*-*β*-d-glucopyranoside [2,8], except for the presence of an acetyl group [*δ*_H_ 2.05 (3H, s, H-8′); *δ*_C_ 20.8 (C-8′) and 172.7 (C-7′)] in it. The positions of the *β*-glucopyranosyl, *p*-coumaroyl, and acetyl groups were confirmed as C-9, C-6, and C-6′, respectively, by HMBC cross peaks of H-9 with C-9″, H-1′ with C-6, and H-6′ with C-7′ (Figure 2). No more signals appeared from the NMR data of compound **2** in methanol-*d*_4_ (Table 1), but considering the molecular formula and fragment peaks, *m/z* 587 [M - malonyl]^+^, 691 [M + H]^+^, 713 [M + Na]^+^, and 1381 [2M + H]^+^, observed in the HRESIMS spectrum of **2**, the presence of an additional malonyl moiety was expected (Figure 5). Therefore, further NMR analysis was performed using a DMSO-*d*_6_ solvent (Table 1). Additional methylene group signals [*δ*_H_ 2.54 (1H, overlapped, H-2′′′) and 2.73 (1H, m, H-2′′′)] were confirmed in the ^1^H NMR and HSQC spectra. The HMBC cross peaks of H-1 with C-1′′′ and H-2′′′ with C-1′′′, as well as the HRESIMS fragment data, indicated that the malonyl moiety is linked with the C-1 position (Figure 2 and Figure 5). The NOESY correlation peaks of H-1 with H-5, H-5 with H-6/H-7, H-7 with H-5/H-9, H-9 with H-5/H-7, and H-14 with H-2*β*/H-8*β* indicated that compound **2** has the same relative configuration with the analogue [2,8] (Figure 3) (HRESIMS and NMR spectra of 2: Supplementary Figures S8–S19). Additionally, (1*R*,5*S*,6*R*,7*S*,9*S*,10*R*)-absolute configuration of **2** was determined by means of ECD study (Figure 4). Thus, compound **2** (askoseoside B) was established as (1*R*,5*S*,6*R*,7*S*,9*S*,10*R*)-1-*O*-malonyl-9-*O*-(*E*-*p*-coumaroyl)-1,6,9-trihydroxy-eudesm-3-ene-6-*O*-(6’-*O*-acetyl)-*β*-d-glucopyranoside.

The molecular formula, C_21_H_38_O_8_, of compound **3** was deduced from the HRESIMS. Signals for four methyl groups, [*δ*_H_ 0.90 (H-12), 1.01 (H-13), 1.28 (H-14), and 1.35 (H-15)]; four methylene groups, [*δ*_H_ 1.01 (H-9*β*), 1.42 (H-3*α*), 1.45 (H-2*α*), 1.62 (H-8*α*), 1.67 (H-3*β*), 1.72 (H-8*β*), 1.88 (H-9*α*), and 1.91 (H-2*β*)]; and a *β*-glucopyranosyl unit were observed in the ^1^H NMR spectrum. An oxygenated quaternary carbon signal [*δ*_C_ 73.5 (C-4)] was detected in the ^13^C NMR data (Table 2). The linkage between glucopyranose and aglycone was identified from the HMBC peaks of H-1′ with C-6 and the positions of each hydroxyl groups were assigned using COSY and HMBC correlation peaks (Figure 2). The planar structure of **3** was confirmed to be the identical as that of ophiopogonoside A [28] and pumilaside A [29], as previously reported. The NOESY correlation peaks of H-1 with H-5, H-5 with H-6/H-7/H-15, H-6 with H-15, and H-14 with H-2*β*/H-8*β* revealed the relative configuration of **3**, which was different from the ophiopogonoside A and pumilaside A (Figure 2). It has been reported that the anomeric carbon signal of secondary alcoholic *β*-d-glucosides depends on the alcohol configuration [30]. In case of eudesmane-type sesquiterpene, the C-1′ resonances of 6*-O*-*β*-d-glucosides indicated that 6*R*-alcohols (*δ*_C_ 104~105) is more deshielded than 6*S*-alcohols (*δ*_C_ 100) in methanol-*d*_4_ [8,31]. Thus, compound **3** (askoseoside C) was proposed as (1*R*,4*S*,5*S*,6*R*,7*S*,10*R*)-1,4,6-trihydroxy-eudesmane-6-*O*-*β*-d-glucopyranoside (HRESIMS and NMR spectra of 3: Supplementary Figures S20–S26).

The molecular formula, C_27_H_48_O_12_, of compound **4** was deduced by HRESIMS. The 1D NMR spectra of compound **4** were similar to those of compound **3**, except for an additional *α*-rhamnopyranosyl unit [anomeric proton: *δ*_H_ 4.82 (1H, s, H-1″)] present in it (Table 2). The linkages of glucopyranosyl and rhamnopyranosyl units with the aglycone were confirmed from the HMBC correlation peaks of H-1′ with C-6 and H-1″ with C-1, respectively (Figure 2). The relative configuration of aglycone in compound **4** was found to be identical to that compound **3** by the NOESY spectrum (Figure 3). Regarding the above-mentioned data and the biogenetic relationship, compound **4** (askoseoside D) was proposed as (1*R*,4*S*,5*S*,6*R*,7*S*,10*R*)-1,4,6-trihydroxy-eudesmane-1-*O*-α-l-rhamnopyranoside-6-*O*-*β*-d-glucopyranoside (HRESIMS and NMR spectra of 4: Supplementary Figures S27–S33).

The structures of the 18 known compounds were assigned as (1*R*,5*S*,6*R*,7*S*,9*S*,10*R*)-1,6,9-trihydroxy-eudesm-3-ene-6-*O*-*β*-d-glucopyranoside (**5**) [8], (1*R*,5*S*,6*R*,7*S*,9*S*,10*R*)-1,6,9,11-tetrahydroxy-eudesm-3-ene-6-*O*-*β*-d-glucopyranoside (**6**) [8], (1*R*,5*S*,6*R*,7*S*,9*S*,10*S*)-1,6,9-trihydroxy-eudesm-3-ene-1,6-di-*O*-*β*-d-glucopyranoside (**7**) [8], 1*β*,4*β*,13-trihydroxy-trans-eudesm-6-ene-1-*O*-*β*-d-glucopyranoside (**8**) [32], apigenin (**9**) [33], quercetin-3-*O*-*β*-d-glucopyranoside (**10**) [34], isorhamnetin-3-*O*-*β*-d-glucopyranoside (**11**) [35], quercetin-3-*O*-*β*-d-rutinoside (**12**) [36], isorhamnetin-3-*O*-*β*-d-rutinoside (**13**) [37], apigenin-7-*O*-*β*-d-glucuronopyranoside (**14**) [38], apigenin-7-*O*-*β*-d-glucuronide methylester (**15**) [39], gymnasterkoreaside A (**16**) [40], gymnasterkoreayne G (**17**) [41], gymnasterkoreayne E (**18**) [41], gymnasterkoreayne B (**19**) [9], gymnasterkoreayne C (**20**) [9], 2(*E*),9(*Z*),16-heptadecatriene-4,6-diyne-8-ol (**21**) [41], and 1-(3′,4′-dihydroxycinnamoyl) cyclopentane-2,3-diol (**22**) [42]. Through this study, two flavonoids (**12** and **15**) and a phenylpropanoid derivative (**22**) are reported for the first time as constituents of *A. koraiensis*. Six compounds (**7**, **8**, **13**, **16**, **20**, and **21**) have not previously been reported as constituents of the flower of this plant. Furthermore, the flavonol glucuronides (**14**−**15**) are specific components found only, to date, in the flowers of this plant.

### 2.2. Effect of Isolated Compounds on EGF- and TPA-Induced Cell Transformation

**Figure 6:**
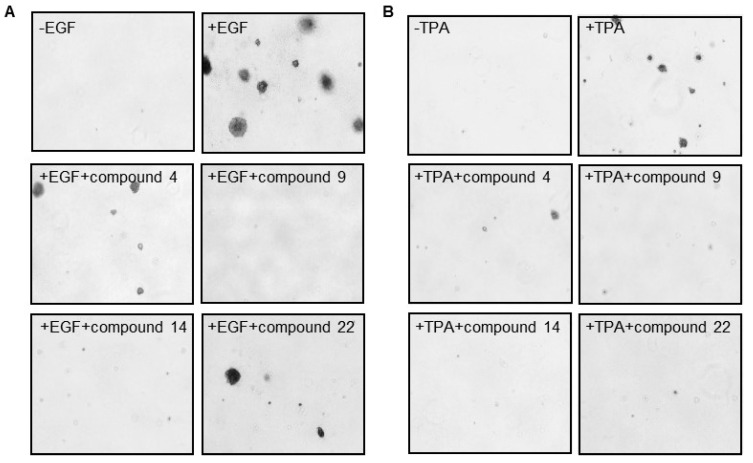
Effects of compounds on (**A**) EGF- or (**B**) TPA-induced cell transformation. Colony growth was assessed by soft agar assay in JB6 Cl41 cells. A and B depict representative images of colony growth in soft agar two weeks following treatment of DMSO, EGF-induced, or TPA-induced cells, respectively, with each of the four compounds.

The cell transformation soft agar assay has been used in evaluating the efficacy of various compounds for development and screening of new anticancer agents by quantifying the proliferation of anchorage-independent cells [26]. To identify the biological effects of the isolated compounds (**1**–**22**), we performed the EGF- and TPA-induced cell transformation assay using the mouse epithelial cell line, JB6 Cl41 (Table 3 and Figure 6). JB6 CI41 cells are widely used to study the molecular mechanisms of tumor promotion and anti-tumor drugs. In addition, both TPA and EGF are well-known tumor promoters that are useful for studying malignant cell transformation [26]. Each compound was used at a concentration of 50 μM and the percentage growth inhibition of cells was determined by comparison with that of the controls (treatment with EGF or TPA alone). Among the 22 compounds, compounds **2**, **4**, **9**, **10**–**15**, **17**, **18**, and **22** inhibited EGF-induced colony growth, while compounds **1**, **3**–**5**, **6**, **9**, **11**, **13**, **14**–**18**, and **22** inhibited TPA-induced colony growth by more than 50%, respectively (Table 3). Further, compounds **4**, **9**, **11**, **13**–**15**, **17**, **18**, and **22** displayed significant inhibition of both EGF- and TPA-induced colony growth (Figure 6), of which askoseoside D (**4**, EGF: 57.8%; TPA: 67.1%), apigenin (**9**, EGF: 88.6%; TPA: 80.2%), apigenin-7-*O*-*β*-d-glucuronopyranoside (**14**, EGF: 79.2%; TPA: 70.7%), and 1-(3′,4′-dihydroxycinnamoyl) cyclopentane-2,3-diol (**22**, EGF: 60.0%; TPA: 72.1%) displayed higher potent activities than that of others. We demonstrated that the isolated compounds significantly inhibited EGF- and TPA-induced cell transformation. These results indicate that constituents of the flowers of *A. koraiensis* could exert anticancer effects by preventing EGF- and TPA-induced tumorigensesis. In previous studies, flavonoids, apigenin (**9**) and its derivatives, isolated from the flowers of *A. koraiensis* showed cytotoxicity to several cancer cells, A549, SK-OV-3, SK-MEL-2, and HCT15 [43]. Apigenin (**9**) and its derivatives are well-known natural bioactive substances obtained from many plant sources and have been reported to have anticancer, antidiabetic, antioxidant, and antiviral effects. In particular, these compounds have been reported to exert a broad-spectrum of anticancer effects against several types of cancer, such as liver, lung, breast, colorectal, and prostate cancers [44]. Polyacetylenes isolated from the roots of this plant, including gymnasterkoreayne B (**19**) and gymnasterkoreayne C (**20**), showed significant cytotoxicity to L1210 tumor cells [9]. On the other hand, gymnasterkoreayne B (**19**), a major component isolated from *A. koraiensis*, has been reported to have an antioxidant effect [45], and also gymnasterkoreayne E (**18**) and gymnasterkoreayne B (**19**) isolated from the aerial parts of this plant have been reported to have cholesterol modulatory activity [32].

Among the compounds tested in this study, apigenin (**9**) and apigenin-7-*O*-*β*-d-glucuronopyranoside (**14**) showed potent anti-carcinogenic effects, and other flavonoids (**10**–**13** and **15**) also showed significant effects. Further, polyacetylenes (**16**–**18**) showed significant anti-carcinogenic effects. These results suggest that various components of *A. koraiensis* may have both cytotoxic activities to cancer cells and anti-carcinogenic effects. Therefore, we suggest that the flower of this plant and its active compounds have potential as anticancer agents or chemo-preventive agents against cancer.

### 2.3. Cytotoxicity of Compounds on NHDF Cell

**Figure 7:**
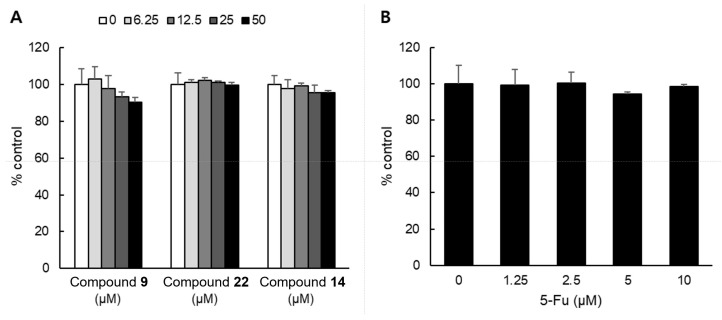
Cytotoxicity of compounds **9**, **14**, and **22** in NHDF cells. NHDF cells were treated (**A**) various concentrations of each compound (0 to 50 μM) and (**B**) various concentrations of 5-Fu (0 to 10 μM). Cell growth was determined by WST-8 assay.

We determined the cytotoxicity of the representative compounds (**9**, **14**, and **22**) in different concentrations, ranging from 0 to 50 μM in a normal cell line NHDF, using the WST-8 assay. The new compound **4** could not be tested due to an insufficient amount available to be evaluated. As shown in Figure 7, after 48 h of treatment with 50 μM of compounds **9**, **14**, and **22**, NHDF cell viabilities were 90.4%, 99.8%, and 95.4%, respectively. The results indicated that the cell viability of the NHDF was not significantly inhibited, and was maintained above 90% at a concentration of 50 µM for each compound during the 48 h. Compared to 5-Fu, an approved chemotherapeutic agent used as a positive control, it was confirmed that the compounds had no effect on the cell viability of NHDF.

## 3. Materials and Methods

### 3.1. General Experimental Procedures

Optical rotations and UV spectra were measured using a P-2000 digital polarimeter (JASCO, Easton, MD, USA) and an Optizen POP spectrophotometer (Mecasys, Daejeon, Republic of Korea), respectively. The ECD spectra were recorded on a JASCO J-1100 spectropolarimeter (JASCO, Easton, MD, USA). NMR spectra were acquired on a DD2 600 MHz FT NMR (Agilent Technologies, Santa Clara, CA, USA) or an AVANCE Ⅲ HD 700 MHz cryogenic NMR spectrometer (Bruker, Billerica, MA, USA). HRESIMS and UPLC were carried out on a Q-TOF micromass spectrometer (Waters, Milford, MA, USA) and Waters Acquity H Class Plus UPLC system using HPLC grade solvent (J.T. Baker, Phillipsburg, NJ, USA), respectively. TLC was performed on RP-18 F_254S_ and silica gel 60 F_254_ pre-coated plates (Merck, Darmstadt, Germany). Chromatographic isolation was carried out via an Isolera^TM^ One and Selekt flash chromatography system (Biotage, Uppsala, Sweden) with Biotage SNAP Ultra (10, 25, or 100 g) and SNAP Ultra C_18_ 120 g pre-packed cartridges, as well as Biotage SNAP dry load cartridges (10, 25, 100, or 340 g scales) manually packed with Sephadex LH-20 (Merck) and Diaion HP-20 (Supelco, Bellefonte, PA, USA) gels.

### 3.2. Plant Material

Dried flowers of *A. koraiensis* were obtained and identified from the National Institute of Forest Science in 2019. A voucher specimen (No. Gyko-19-1903) was deposited at the Herbal Medicine Resources Research Center, KIOM, Republic of Korea.

### 3.3. Extraction and Isolation

The dried flowers of *A. koraiensis* (129.0 g) were ground and extracted with 70% EtOH (3 L × 3 times), and then evaporated to obtain the total extract (47.2 g, 36.6%). A total of 45 g of the extract was used for isolation without partitioning. Chromatographic isolation was performed by a flash chromatography system (MPLC). The extract was separated on a Diaion HP-20 gel in a 340 g scale cartridge (D.W./MeOH, 100:0 to 0:100) to produce 12 fractions (F01−F12); following which, compound **14** (427.0 mg) was obtained from F05 by precipitation.

F03 (3.1 g) was fractionated using two C_18_ 120 g cartridges (D.W./MeOH, 100:0 to 50:50) to yield 12 subfractions (F0301−F0312). F0305 (202.2 mg) was further fractionated on a Sephadex LH-20 gel column (two 100 g scale cartridges, D.W./MeOH, 100:0) to produce compound **22** (66.5 mg).

F06 (2.0 g) was separated using two C_18_ 120 g cartridges (D.W./MeOH, 90:10 to 40:60) to obtain 16 subfractions (F0601−F0616). F0610 (340.5 mg) was fractionated on a Sephadex LH-20 gel column (two 100 g scale cartridges, D.W./MeOH, 100:0 to 40:60) to obtain 9 subfractions (F061001−F061009). Compounds **6** (48.0 mg) and **8** (2.7 mg) were obtained from F061002 (63.2 mg) using two silica 25 g cartridges (CHCl_3_/MeOH/add D.W., 80:20:2 to 70:30:5). F061003 (77.2 mg) was separated using two silica 25 g cartridges (CHCl_3_/MeOH/add D.W., 80:20:2) to yield compound **16** (36.6 mg). Separation of compounds **12** (19.2 mg) and **10** (24.7 mg) from F061006 (44.5 mg) and F061008 (53.4 mg), respectively, which was performed using three silica 10 g cartridges (CHCl_3_/MeOH/add D.W., 80:20:2 to 70:30:5). Fractionation of F0611 (340.2 mg) was performed on a Sephadex LH-20 gel column (two 100 g scale cartridges, D.W./MeOH, 100:0 to 40:60), followed by two silica 25 g cartridges (CHCl_3_/MeOH/add D.W., 80:20:2 to 70:30:5) to produce compound **7** (99.8 mg). Compounds **13** (11.1 mg) and **11** (7.5 mg) were obtained from F061306 (23.4 mg) and F061307 (44.1 mg), respectively, using two silica 10 g cartridges (CHCl_3_/MeOH/add D.W., 80:20:2). F0615 (105.5 mg) was separated using a Sephadex LH-20 gel column (two 25 g scale cartridges, D.W./MeOH, 100:0 to 20:80) to produce compound **15** (4.6 mg) and five subfractions (F061501−F061505). F061502 (67.3 mg) was fractionated by two silica 25 g cartridges (CHCl_3_/MeOH/add D.W., 80:20:2 to 70:30:5) to obtain compounds **1** (1.8 mg), **3** (3.5 mg), **4** (3.3 mg), and **5** (38.1 mg).

F09 (3.4 g) was separated using two C_18_ 120 g cartridges (D.W./MeOH, 50:50 to 0:100) to yield 21 fractions (F0901−F0921) Thereafter, compound **9** (31.1 mg) was obtained by precipitation from F0904. F0903 (19.0 mg) was firstly separated using two silica 10 g cartridges (CHCl_3_/MeOH/add D.W., 90:10:1 to 80:20:2) and then on Sephadex LH-20 gel cartridges (four 10 g scale cartridges, D.W./MeOH, 80:20 to 70:30) to produce compound **2** (3.5 mg). Separation of compound **17** (11.6 mg) from F0907 (237.1 mg) was performed on Sephadex LH-20 gel cartridges (four 10 g scale cartridges, D.W./MeOH, 60:40 to 0:100). Fractionation of F0913 (206.2 mg) was performed using a silica 100 g cartridge (CHCl_3_/MeOH/add D.W., 95:5:0.5 to 70:30:5) to give compound **18** (15.0 mg). Compound **19** (8.0 mg) was obtained from F0915 (84.4 mg) using two silica 25 g cartridges (CH_2_Cl_2_, 100%). F0916 (33.7 mg) was purified using three silica 10 g cartridges (*n*-hexane/EtOAc, 100:0 to 80:20) to produce compound **20** (10.1 mg). Chromatographic separation of F0918 (23.6 mg) was performed by two silica 10 g cartridges (CHCl_3_, 100%) to produce compound **21** (3.0 mg). The purity of all isolated compounds was greater than 98%.

#### 3.3.1. Askoseoside A (Compound **1**)

Amorphous powder; [α]^22^_D_ –10 (*c* 0.05, MeOH); UV (MeOH) λ_max_ (log *ε*) 205 (3.47), 249 (3.63) nm; ECD (*c* 0.5 mM, MeOH) Δ*ε* +10.1 (205), +0.2 (226), +8.4 (252), –1.9 (326); see Table 1 for ^1^H (methanol-*d*_4_, 600 MHz) and ^13^C (methanol-*d*_4_, 150 MHz) NMR data; HRESIMS *m/z* 453.2093 [M + Na]^+^ (calculated for C_21_H_34_O_9_Na, 453.2101).

#### 3.3.2. Askoseoside B (Compound **2**)

Amorphous powder; [α]^22^_D_ 22.7 (*c* 0.1, MeOH); UV (MeOH) λ_max_ (log *ε*) 208 (3.84), 228 (3.75), 314 (3.99) nm; ECD (*c* 0.5 mM, MeOH) Δ*ε* +7.7 (220), –8.2 (250), +4.4 (302); see Table 1 for ^1^H (methanol-*d*_4_, 600 MHz), ^1^H (DMSO-*d*_6_, 700 MHz), ^13^C (methanol-*d*_4_, 150 MHz), and ^13^C (DMSO-*d*_6_, 175 MHz) NMR data; HRESIMS *m/z* 691.2969 [M + H]^+^ (calculated for C_35_H_47_O_14_, 691.2966).

#### 3.3.3. Askoseoside C (Compound **3**)

Amorphous powder; [α]^22^_D_ –11 (*c* 0.1, MeOH); UV (MeOH) λ_max_ (log *ε*) 207 (3.73) nm; see Table 2 for ^1^H (methanol-*d*_4_, 600 MHz) and ^13^C (methanol-*d*_4_, 150 MHz) NMR data; HRESIMS *m*/*z* 441.2462 [M + Na]^+^ (calculated for C_21_H_38_O_8_Na, 441.2464).

#### 3.3.4. Askoseoside D (Compound **4**)

Amorphous powder; [α]^22^_D_ –9 (*c* 0.1, MeOH); UV (MeOH) λ_max_ (log *ε*) 209 (3.98) nm; see Table 2 for ^1^H (methanol-*d*_4_, 600 MHz) and ^13^C (methanol-*d*_4_, 150 MHz) NMR data; HRESIMS *m/z* 587.3036 [M + Na]^+^ (calculated for C_27_H_48_O_12_Na, 587.3043).

### 3.4. Acid Hydrolysis and Sugar Identification

Sugar units of new compounds **1**−**4** (each 0.5 mg) were confirmed according to the method of Tanaka et al. [46,47]. The standards (l-glucose, d-glucose, l- rhamnose, and d-rhamnose; each 1.0 mg, Merck) and hydrolyzed compounds **1**−**4** were reacted and then analyzed using a UPLC system. A Waters CSH C_18_ analytical column with 30% acetonitrile isocratic elution (0.05% formic acid in D.W./acetonitrile, 0.3 mL/min, 10 min, 250 nm) was carried out for analysis. Standard derivatives were recorded at *t*_R_ 4.056 min (l-glucose derivative), *t*_R_ 4.427 min (d-glucose derivative), *t*_R_ 3.950 min (d-rhamnose derivative), and *t*_R_ 7.307 min (l-rhamnose derivative).

### 3.5. Computational Methods

Conformer distribution, optimization, and ECD analysis were performed, as described previously [48]. All conformers proposed in the study were found using the Spartan’14 (Wave-function, Inc., Irvine, CA, USA). The conformers were subjected to geometry optimization using the Gaussian’09 (Gaussian, Inc., Wallingford, CT, USA) in the DFT [B3LYP functional/6-31+G(d,p) basis set] level, and ECD calculations were performed at the TDDFT(CAM-B3LYP/SVP basis set) level with a CPCM solvent model in MeOH.

### 3.6. EGF- or TPA-Induced Cell Transformation (Soft Agar) Assay

To evaluate the cellular anchorage-independent growth, the soft agar colony formation assay was conducted according to modified protocol [26]. A mouse epithelial JB6 Cl41 cell was obtained from the ATCC (Manassas, VA, USA) and cultured in Eagle’s minimum essential medium (MEM; GIBCO, Invitrogen GmbH, Karlsruhe, Germany) supplemented with 5% fetal bovine serum (FBS; GIBCO), 100 U/mL penicillin, and 100 μg/mL streptomycin (GIBCO). Basal Medium Eagle (BME; Sigma Aldrich, St. Louis, MO, USA) supplemented with 10% FBS, 2 mmol/L L-glutamine, and 25 μg/mL gentamicin was mixed with 0.6% agar containing DMSO, EGF (10 ng/mL) or TPA (10 ng/mL), and/or each compound (50 μM) and solidified as the bottom agar of 6-well plates. JB6 Cl41 cells (8000 cells/well) were suspended in 1 mL of BME medium supplemented with 0.3% agar containing DMSO, EGF (10 ng/mL) or TPA (10 ng/mL) and/or each compound (**50** μM) and added to the bottom agar layer. The plates were incubated (37 °C) in a 5% CO_2_ incubator for two weeks. Colonies were visualized by a microscope (Leica Microsystems, Germany) and the numbers analyzed using an Image-Pro Plus software ver.6.1 (Media Cybernetics, Rockville, MD, USA).

### 3.7. WST-8 Assay

To estimate cytotoxicity of compounds, normal human dermal fibroblast (NHDF; ATCC) cells (5000 cells/well) were seeded into each well of 96-well plates. After 24 h incubation, the cells were treated with various concentration of the representative compounds **9**, **14**, **22**, and 5-fluorouracil (5-Fu; Sigma Aldrich, St. Louis, MO, USA) for 48 h. The cytotoxicity of each compound was measured using Quanti-MAX WST-8 Cell Viability Assay Kit reagent (Biomax, Seoul, Republic of Korea). According to the instructions, the absorbance was measured at 450 nm using the Multiskan SkyHigh Spectrophotometer (Thermo Scientific, Vantaa, Finland).

## 4. Conclusions

In the current study, four new compounds (**1**–**4**) were isolated from the flowers of *A. koraiensis* and their structures were identified through spectroscopic studies. The anticancer activity of a total of 22 compounds (**1**–**22**) isolated in this study was evaluated by cell transformation assay, and most of them showed significant anti-carcinogenic activity. In particular, four compounds, askoseoside D (**4**), apigenin (**9**), apigenin-7-*O*-*β*-d-glucuronopyranoside (**14**), and 1-(3′,4′-dihydroxycinnamoyl) cyclopentane-2,3-diol (**22**), including new compound (**4**), showed higher activity than other compounds. In addition, compounds **9**, **14**, and **22** did not exhibit any toxic effects in the cell viability assay for NHDF, which is a normal skin cell line. This study not only expands the chemical composition of *A. koraiensis*, but also provides new information on their physiological activities. In addition, this study suggests the potential value of this plant and its active compounds as natural anticancer agents.

## Figures and Tables

**Table 1:** ^1^H and ^13^C NMR data of compounds **1** and **2**.

	1 *^a,e^*		2 *^a,e^*		2 *^b,f^*	
Position	*δ*_C_, Type	*δ*_H_ Multi (*J* in Hz)	*δ*_C_, Type	*δ*_H_ Multi (*J* in Hz)	*δ*_C_, Type	*δ*_H_ Multi (*J* in Hz)
1	77.1, CH	3.99, dd (12.9, 4.7)	80.5, CH	4.89, dd (10.0, 5.9)	76.9, CH	4.68, dd (9.7, 6.2)
2	42.8, CH_2_	2.45, dd (15.8, 4.7), 2.66, dd (16.4, 12.9)	29.7, CH_2_	2.090, m *^c^*, 2.25, m	28.5, CH_2_	1.89, m, 2.07, m
3	200.8, C		120.4, CH	5.26, br d (2.3)	118.9, CH	5.20, br s
4	134.1, C		135.4, C		133.9, C	
5	159.0, C		52.2, CH	2.093, s	49.9, CH	2.09, s
6	78.23, CH	4.89, br d (1.8)	75.0, CH	4.47, s	72.1, CH	4.36, s
7	48.4, CH	1.04, m *^c^*	51.4, CH	1.17, m	49.1, CH	1.16, m
8	29.5, CH_2_	1.78, m, 1.86, m *^c^*	28.9, CH_2_	1.91, m	27.3, CH_2_	1.70, m, 184, m
9	81.7, CH	3.65, dd (11.7, 4.7)	80.3, CH	4.97, dd (10.6, 5.9)	77.7, CH	4.83, dd (11.4, 4.8)
10	46.7, C		42.2, C		40.2, C	
11	28.8, CH	2.03, m	29.2, CH	1.94, m	27.2, CH	1.90, m
12	21.47, CH_3_	0.99, d (6.5)	21.5, CH_3_	1.02, d (6.5)	20.9, CH_3_	0.95, d (6.5)
13	21.51, CH_3_	1.03, d (6.5)	21.8, CH_3_	0.92, d (6.5)	21.4, CH_3_	0.84, d (6.5)
14	12.3, CH_3_	1.36, s	11.6, CH_3_	1.36, s	10.7, CH_3_	1.22, s
15	11.8, CH_3_	1.85, s	21.6, CH_3_	1.83, s	20.4, CH_3_	1.78, s
1′	106.7, CH	4.37, d (7.6)	103.7, CH	4.40, d (7.6)	101.7, CH	4.27, d (7.3)
2′	75.9, CH	3.16, (8.2)	76.1, CH	3.15, td (7.0, 2.3)	74.3, CH	2.93, m
3′	78.17, CH	3.33, m *^c^*	78.3, CH	3.308, m *^c^*	76.8, CH	3.11, t (8.6)
4′	71.7, CH	3.28, t (8.8)	71.5, CH	3.310, m *^c^*	70.1, CH	3.07, t (9.2)
5′	77.9, CH	3.12, m	74.9, CH	3.36, m	73.4, CH	3.26, ddd (9.2, 6.8, 1.9)
6′	62.8, CH_2_	3.60, m	64.7, CH_2_	4.14, dd (11.7, 5.3), 4.41, dd (11.7, 1.8)	63.6, CH_2_	3.99, dd (11.6, 6.8), 4.28, m *^c^*
7′			172.7, C		170.2, C	
8′			20.8, CH_3_	2.05, s	20.6, CH_3_	1.99, s
1″			127.3, C		124.9, C	
2″			131.3, CH	7.47, d (8.8)	130.1, CH	7.51, d (8.8)
3″			116.9, CH	6.80, d (8.8)	115.8, CH	6.78, d (8.8)
4″			161.3, C		160.0, C	
5″			116.9, CH	6.80, d (8.8)	115.8, CH	6.78, d (8.8)
6″			131.3, CH	7.47, d (8.8)	130.1, CH	7.51, d (8.8)
7″			146.6, CH	7.61, d (15.8)	144.1, CH	7.48, d (15.8)
8″			116.2, CH	6.32, d (15.8)	114.9, CH	6.29, d (15.8)
9″			168.7, C		165.7, C	
1′′′					168.8, C	
2′′′					45.7, CH_2_	2.54, m *^c^*, 2.73, m
3′′′					nt *^d^*	

*^a^* Measured in methanol-*d*_4_. *^b^* Measured in DMSO-*d*_6_. *^c^* Signals partially overlapped. *^d^* not detected. *^e^* Recorded at 600 MHz (^1^H NMR) and 150 MHz (^13^C NMR). *^f^* Recorded at 700 MHz (^1^H NMR) and 175 MHz (^13^C NMR).

**Table 2:** ^1^H and ^13^C NMR data (methanol-*d*_4_) for compounds **3** and **4**.

	3 *^b^*		4 *^b^*	
Position	*δ*_C_, Type	*δ*_H_ Multi (*J* in Hz)	*δ*_C_, Type	*δ*_H_ Multi (*J* in Hz)
1	81.8, CH	3.11, m	85.1, CH	3.17, m *^a^*
2	27.8, CH_2_	1.45, m, 1.91, m	22.8, CH_2_	1.67, m, 1.75, m *^a^*
3	41.0, CH_2_	1.42, m, 1.67, m	40.5, CH_2_	1.38, m, 1.72, m *^a^*
4	73.5, C		73.4, C	
5	55.2, CH	0.99, br s	55.5, CH	1.05, s
6	76.9, CH	4.65, s	76.8, CH	4.65, s
7	54.2, CH	0.85, m	54.1, CH	0.87, m
8	21.7, CH_2_	1.62, m, 1.72, m	21.7, CH_2_	1.62, m, 1.74, m *^a^*
9	41.6, CH_2_	1.01, m, 1.88, m	42.3, CH_2_	1.98, dt (13.4, 2.9), 1.03, m
10	41.1, C		40.8, C	
11	27.5, CH	2.03, m	27.5, CH	2.03, m
12	21.6, CH_3_	0.90, d (6.5)	21.5, CH_3_	0.91, d (6.7)
13	22.5, CH_3_	1.01, d (6.5)	22.4, CH_3_	1.02, d (6.7)
14	14.9, CH_3_	1.28, s	15.9, CH_3_	1.32, s
15	29.8, CH_3_	1.35, s	29.7, CH_3_	1.37, s
1′	105.3, CH	4.48, d (7.6)	105.3, CH	4.48, d (7.6)
2′	76.2, CH	3.17, t (8.2)	76.1, CH	3.16, m *^a^*
3′	78.1, CH	3.32, m *^a^*	78.1, CH	3.32, m *^a^*
4′	71.5, CH	3.33, m *^a^*	71.5, CH	3.33, m *^a^*
5′	78.4, CH	3.34, m *^a^*	78.4, CH	3.34, m *^a^*
6′	62.5, CH_2_	3.70, dd (11.7, 4.7), 3.90, d (11.7)	62.5, CH_2_	3.70, dd (12.4, 5.7), 3.90, d (12.4)
1″			98.0, CH	4.82, s
2″			73.3, CH	3.74, m *^a^*
3″			72.7, CH	3.64, dd (9.5, 2.9)
4″			74.0, CH	3.38, t (9.5)
5″			70.3, CH	3.73, m *^a^*
6″			18.1, CH_3_	1.24, d (5.7)

*^a^* Signals partially overlapped. *^b^* Recorded at 600 MHz (^1^H NMR) and 150 MHz (^13^C NMR).

**Table 3:** Inhibitory activities of compounds on EGF- and TPA-induced cell transformation.

Compounds	Inhibitory Activity % (EGF) *^a^*	Inhibitory Activity % (TPA) *^b^*
**1**	22.5 ± 9.5	51.4 ± 5.6
**2**	60.4 ± 2.0	- *^c^*
**3**	44.2 ± 4.4	52.5 ± 4.5
**4**	57.8 ± 1.5	67.1 ± 6.6
**5**	36.8 ± 7.8	52.2 ± 5.1
**6**	25.4 ± 3.6	58.6 ± 6.3
**7**	37.9 ± 2.7	45.7 ± 6.6
**8**	36.1 ± 9.9	45.8 ± 7.2
**9**	88.6 ± 2.8	80.2 ± 2.3
**10**	56.9 ± 1.2	40.7 ± 6.9
**11**	51.5 ± 2.4	52.0 ± 6.3
**12**	52.6 ± 5.5	36.4 ± 2.8
**13**	64.1 ± 8.3	66.2 ± 4.2
**14**	79.2 ± 4.2	70.7 ± 1.1
**15**	50.0 ± 5.5	58.1 ± 4.7
**16**	27.7 ± 7.2	72.1 ± 1.8
**17**	59.3 ± 3.8	73.7 ± 3.9
**18**	65.5 ± 5.6	66.4 ± 1.6
**22**	60.0 ± 0.6	72.1 ± 1.4

*^a^* Inhibitory effects on EGF-induced cell transformation. *^b^* Inhibitory effects on TPA-induced cell transformation. *^c^* The amount of compound **2** was not sufficient enough to perform the assay. Compounds **19**–**21** were unusable for the assay because of low solubility.

## Data Availability

Not applicable.

## Supplementary Materials

The following supporting information can be downloaded at: https://www.mdpi.com/article/10.3390/plants12081726/s1, Figures S1–S7: HRESIMS, ^1^H NMR, ^13^C NMR, HSQC, HMBC, COSY, and NOESY spectra of compound **1**. Figures S8–S19: HRESIMS, ^1^H NMR (methanol-*d*_4_), ^13^C NMR (methanol-*d*_4_), HSQC (methanol-*d*_4_), HMBC (methanol-*d*_4_), COSY (methanol-*d*_4_), NOESY (methanol-*d*_4_), ^1^H NMR (DMSO-*d*_6_), ^13^C NMR (DMSO-*d*_6_), HSQC (DMSO-*d*_6_), HMBC (DMSO-*d*_6_), and COSY (DMSO-*d*_6_) spectra of compound **2**. Figures S20–S26: HRESIMS, ^1^H NMR, ^13^C NMR, HSQC, HMBC, COSY, and NOESY spectra of compound **3**. Figures S27–S33: HRESIMS, ^1^H NMR, ^13^C NMR, HSQC, HMBC, COSY, and NOESY spectra of compound **4**.
