# Supplementary material for: New Sesquiterpene Glycosides from the Flowers of Aster koraiensis and Their Inhibition Activities on EGF- and TPA-Induced Cell Transformation
Source: Plants (Basel). 2023 Apr 20;12(8):1726. doi: 10.3390/plants12081726 (PMC10146194; doi:10.3390/plants12081726)

Supporting Information for

New Sesquiterpene Glycosides from the Flowers of *Aster koraiensis* and their inhibition activities on EGF- and TPA-induced cell transformation

Young Hye. Seo,[1,‡] *Ji-Young Kim,*[2,‡] *Seung Mok Ryu,*[1] *Sun-Young Hwang,*[3] *Mee-Hyun Lee,*[3] *Nahyun Kim,*[4] *Hojun Son,*[5] *A Yeong Lee,* [1] *Hyo Seon Kim,* [1] *Byeong Cheol Moon,* [1] *Dae Sik Jang,* [2,*] *and Jun Lee*[1,*]

[1]Herbal Medicine Resources Research Center, Korea Institute of Oriental Medicine (KIOM), Naju 58245, Republic of Korea

[2]Department of Biomedical and Pharmaceutical Sciences, Graduate School, Kyung Hee University, Seoul 02447, Republic of Korea

[3]College of Korean Medicine, Dongshin University, Naju 58245, Republic of Korea

[4]Division of Forest Industrial Materials, Department of Forest Products and Industry, National Institute of Forest Science, Seoul 02455, Republic of Korea

[5]Forest Medicinal Resources Research Center (FMRRC), National Institute of Forest Science, Yeongju 36040, Republic of Korea

Figure S1. HRESIMS spectrum of the new compound **1**.

Figure S2. ^{1}H NMR spectrum (600 MHz, methanol-d_4) of the new compound **1**.

Figure S3. ^{13}C NMR spectrum (150 MHz, methanol-d_4) of the new compound **1**.

Figure S4. HSQC spectrum (600 MHz, methanol-d_4) of the new compound **1**.

Figure S5. HMBC spectrum (600 MHz, methanol-d_4) of the new compound **1**.

Figure S6. COSY spectrum (600 MHz, methanol-d_4) of the new compound **1**.

Figure S7. NOESY spectrum (600 MHz, methanol-d_4) of the new compound **1**.

Figure S8. HRESIMS spectrum of the new compound **2**.

Figure S9. ^{1}H NMR spectrum (600 MHz, methanol-d_4) of the new compound **2**.

Figure S10. ^{13}C NMR spectrum (150 MHz, methanol-d_4) of the new compound **2**.

Figure S11. HSQC spectrum (600 MHz, methanol-d_4) of the new compound **2**.

Figure S12. HMBC spectrum (600 MHz, methanol-d_4) of the new compound **2**.

Figure S13. COSY spectrum (600 MHz, methanol-d_4) of the new compound **2**.

Figure S14. NOESY spectrum (600 MHz, methanol-d_4) of the new compound **2**.

Figure S15. ^{1}H NMR spectrum (700 MHz, DMSO-d_6) of the new compound **2**.

Figure S16. ^{13}C NMR spectrum (175 MHz, DMSO-d_6) of the new compound **2**.

Figure S17. HSQC spectrum (700 MHz, DMSO-d_6) of the new compound **2**.

Figure S18. HMBC spectrum (700 MHz, DMSO-d_6) of the new compound **2**.

Figure S19. COSY spectrum (700 MHz, DMSO-d_6) of the new compound **2**.

Figure S20. HRESIMS spectrum of the new compound **3**.

Figure S21. ^{1}H NMR spectrum (600 MHz, methanol-d_4) of the new compound **3**.

Figure S22. ^{13}C NMR spectrum (150 MHz, methanol-d_4) of the new compound **3**.

Figure S23. HSQC spectrum (600 MHz, methanol-d_4) of the new compound **3**.

Figure S24. HMBC spectrum (600 MHz, methanol-d_4) of the new compound **3**.

Figure S25. COSY spectrum (600 MHz, methanol-d_4) of the new compound **3**.

Figure S26. NOESY spectrum (600 MHz, methanol-d_4) of the new compound **3**.

Figure S27. HRESIMS spectrum of the new compound **4**.

Figure S28. ^{1}H NMR spectrum (600 MHz, methanol-d_4) of the new compound **4**.

Figure S29. ^{13}C NMR spectrum (150 MHz, methanol-d_4) of the new compound **4**.

Figure S30. HSQC spectrum (600 MHz, methanol-d_4) of the new compound **4**.

Figure S31. HMBC spectrum (600 MHz, methanol-d_4) of the new compound **4**.

Figure S32. COSY spectrum (600 MHz, methanol-d_4) of the new compound **4**.

Figure S33. NOESY spectrum (600 MHz, methanol-d_4) of the new compound **4**.

Figure S1. HRESIMS spectrum of the new compound **1**.

Tolerance = 5.0 PPM / DBE: min = -1.5, max = 20.0
Element prediction: Off
Number of isotope peaks used for i-FIT = 5

Monoisotopic Mass, Even Electron Ions
199 formula(e) evaluated with 1 results within limits (up to 20 closest results for each mass)
Elements Used:
C: 0-100 H: 0-200 O: 0-50 Na: 0-1
20191216_SeoYH_Gyko7F-J18_pos_re 207 (2.326)
1: TOF MS ES+

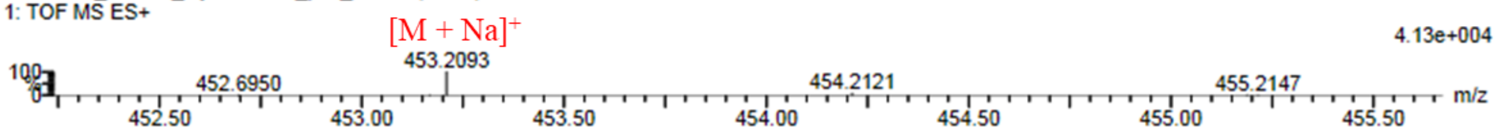

	Mass	RA	Calc. Mass	mDa	PPM	DBE	i-FIT	Norm	Conf(%)	Formula
Minimum:		15.00				-1.5				
Maximum:		100.00		5.0	5.0	20.0				
	453.2093	100.00	453.2101	-0.8	-1.8	4.5	43.0	n/a	n/a	C21 H34 O9 Na

Figure S2. ^{1}H NMR spectrum (600 MHz, methanol-d_4) of the new compound **1**.

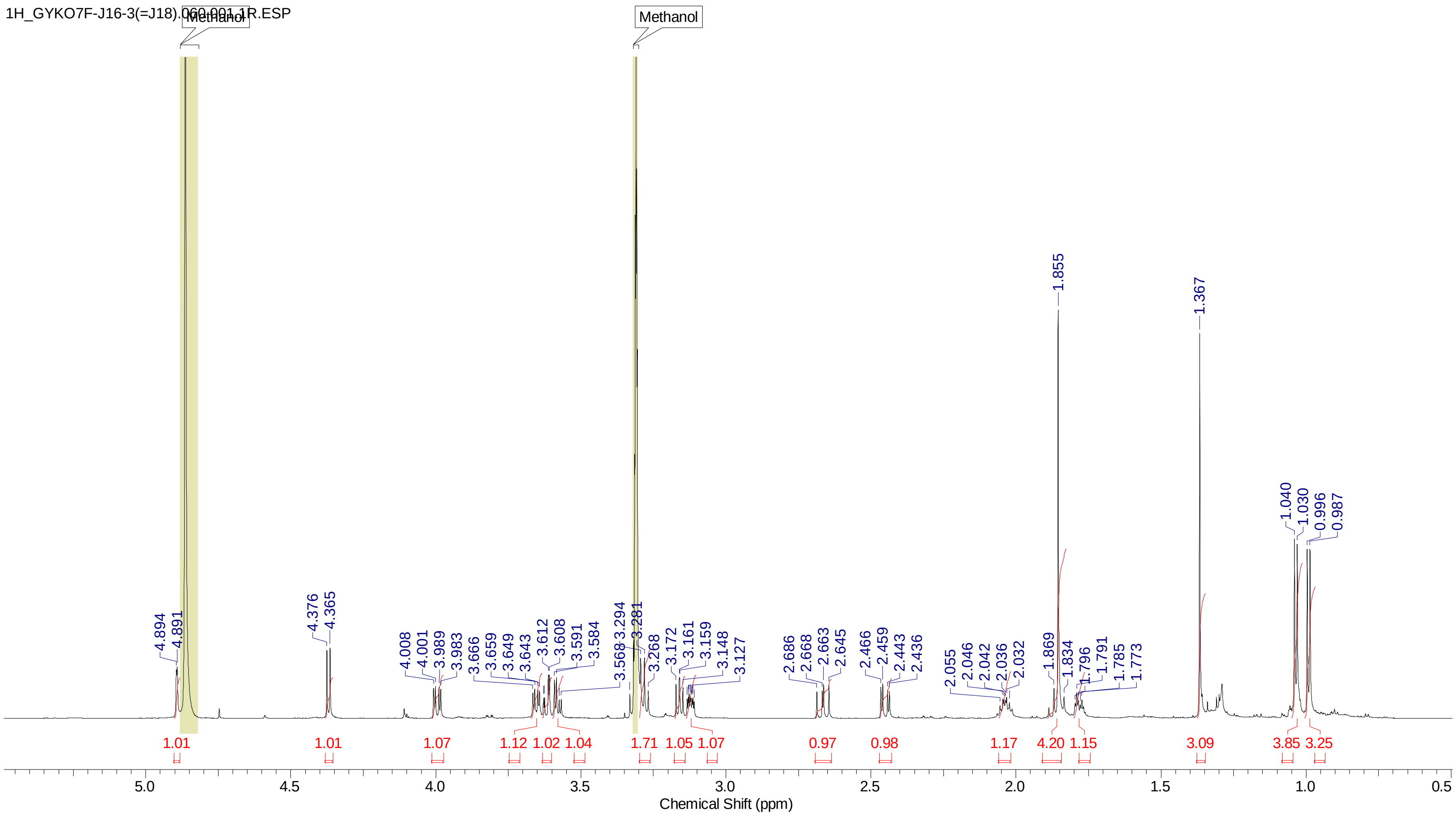

Figure S3. ^{13}C NMR spectrum (150 MHz, methanol-d_4) of the new compound **1**.

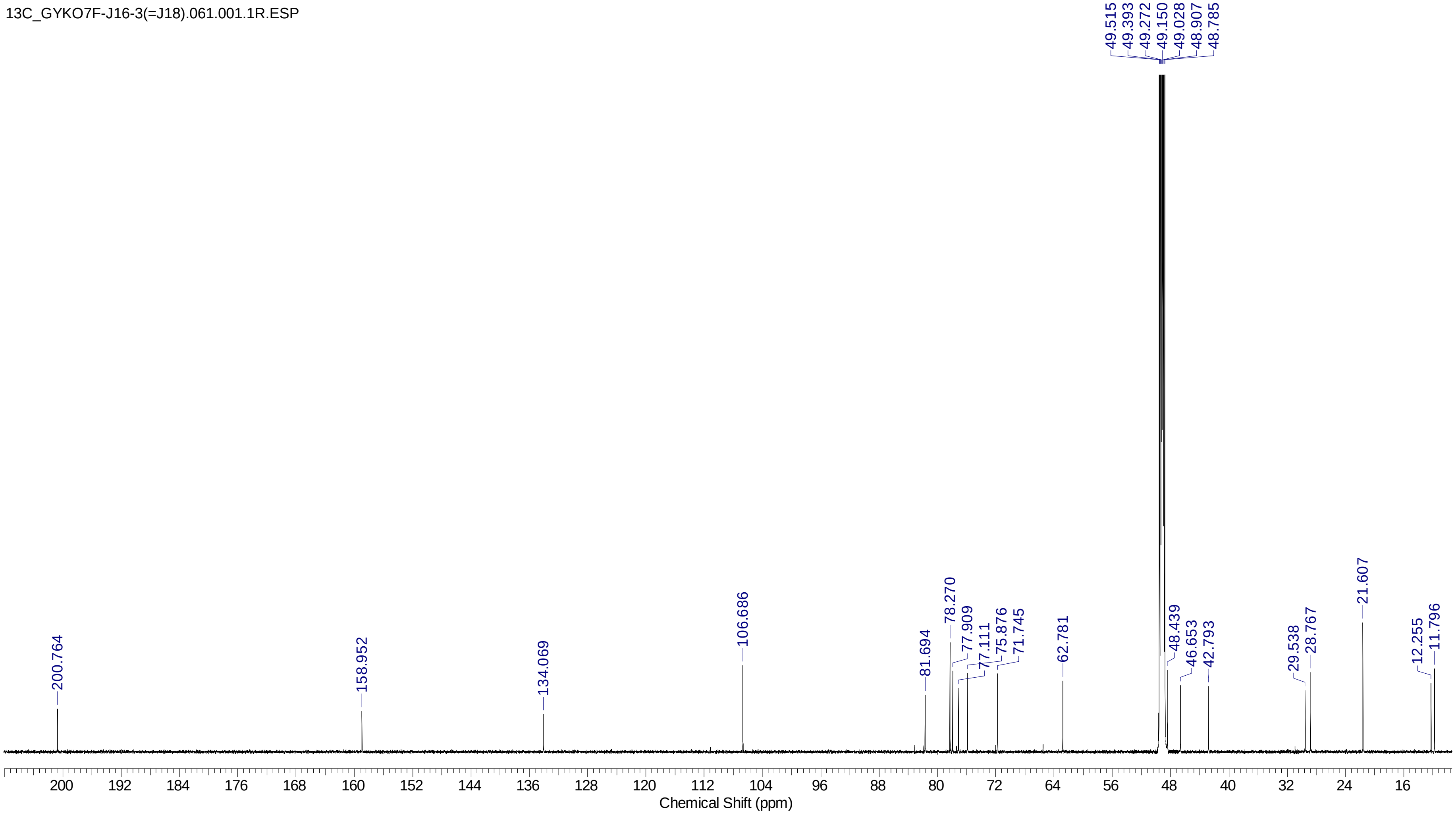

Figure S4. HSQC spectrum (600 MHz, methanol-d_4) of the new compound **1**.

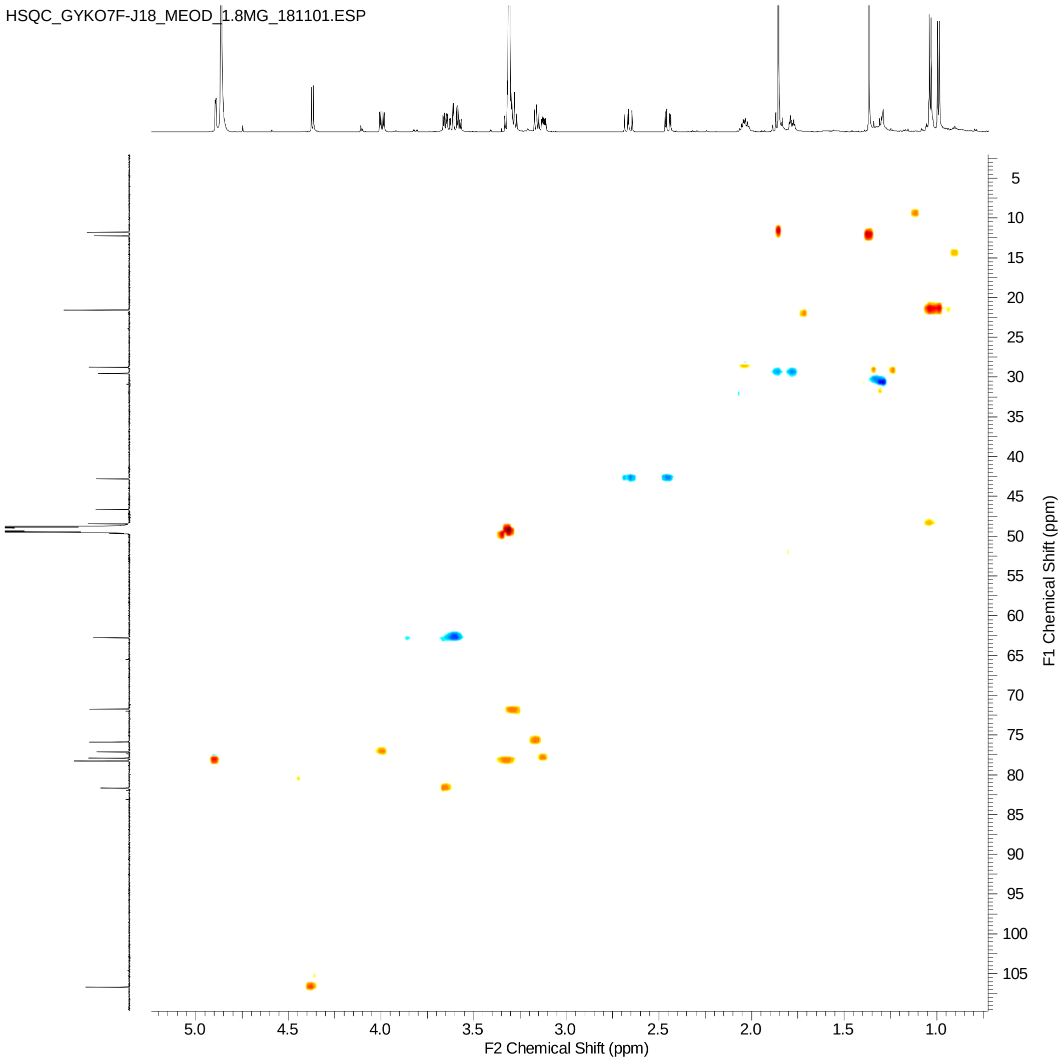

Figure S5. HMBC spectrum (600 MHz, methanol-d_4) of the new compound **1**.

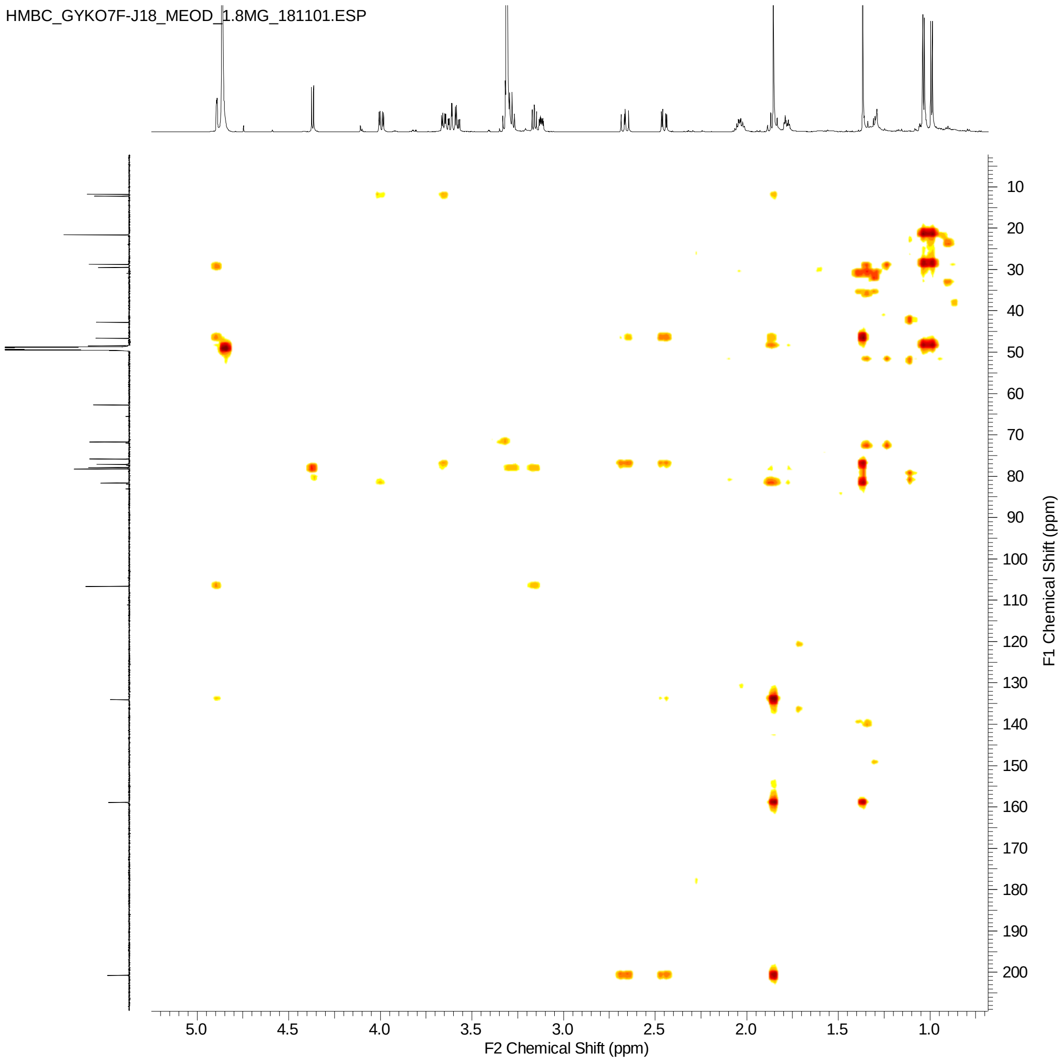

Figure S6. COSY spectrum (600 MHz, methanol-d_4) of the new compound **1**.

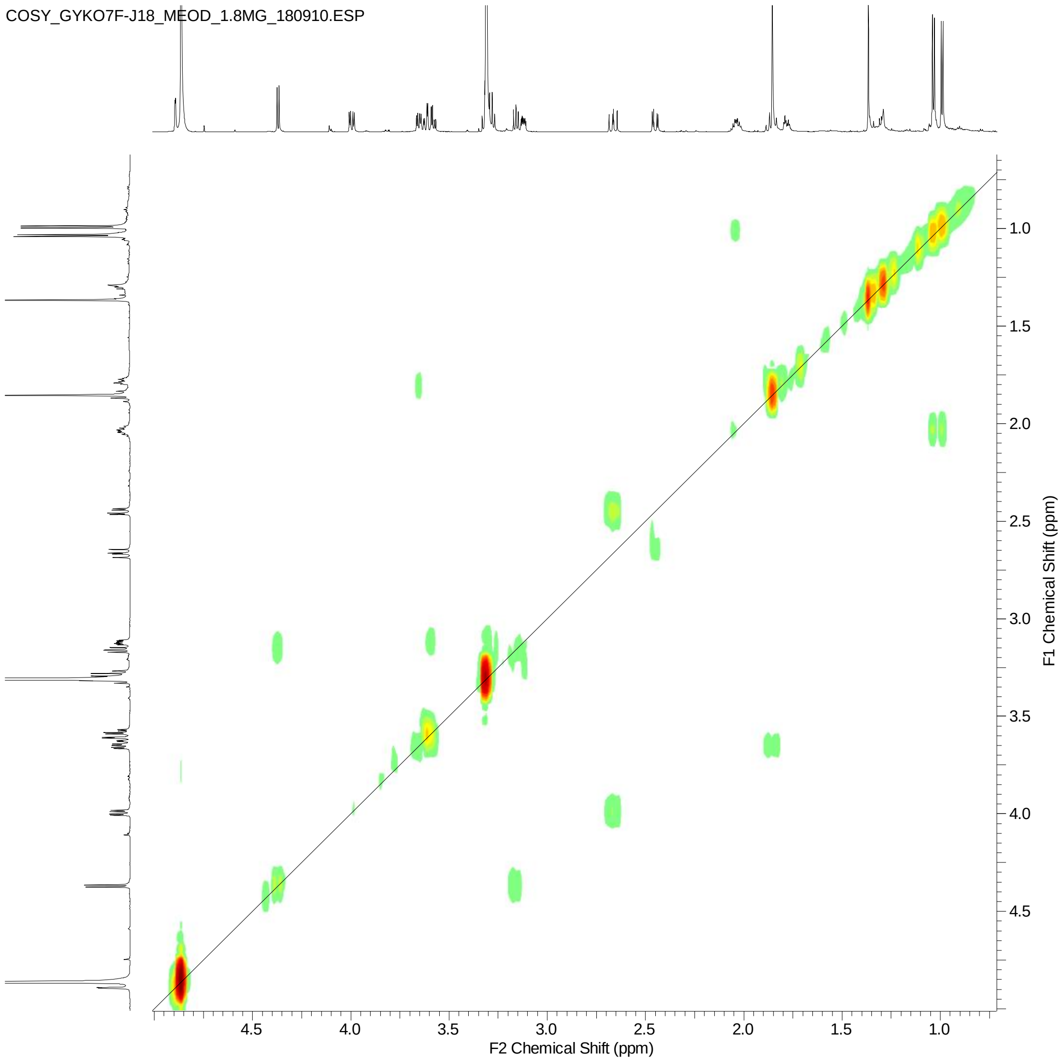

Figure S7. NOESY spectrum (600 MHz, methanol-d_4) of the new compound **1**.

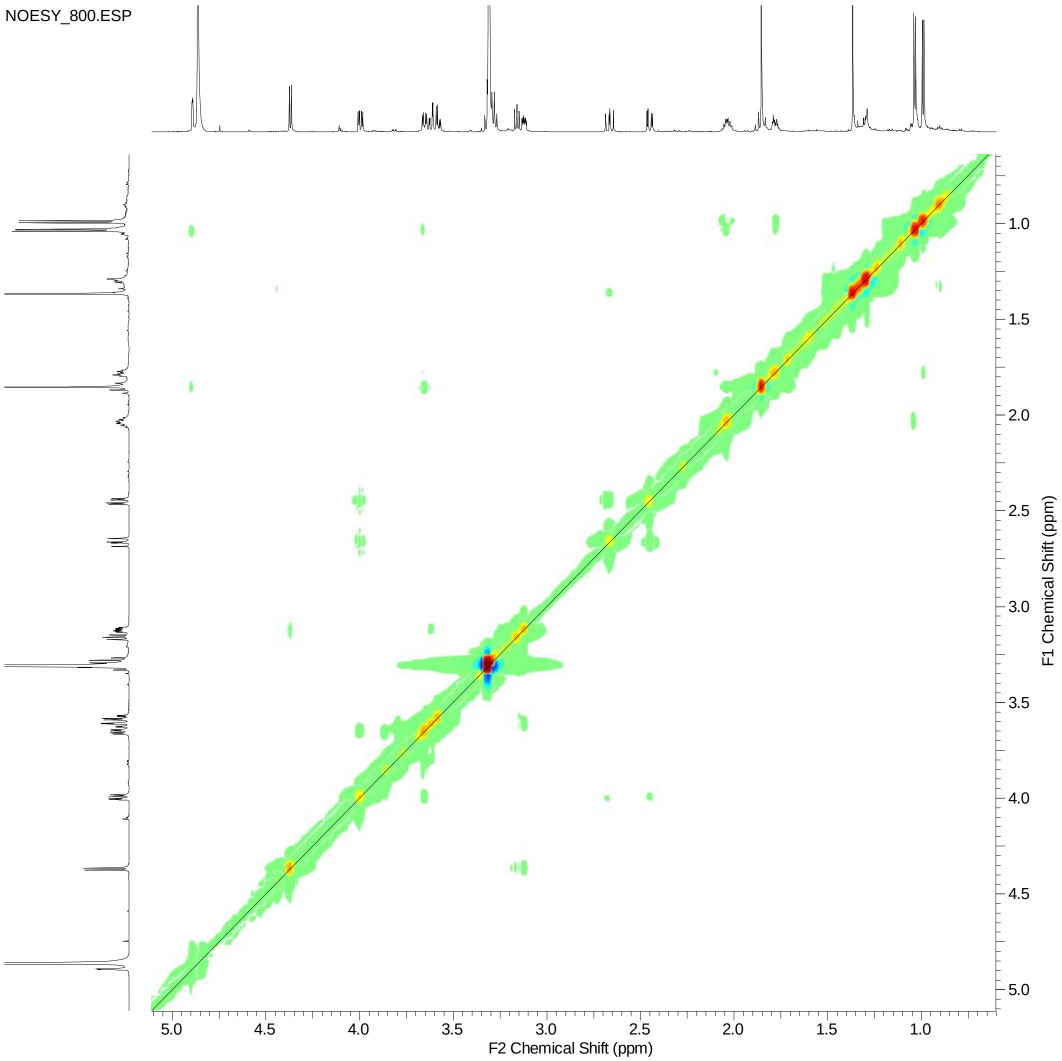

Figure S8. HRESIMS spectrum of the new compound **2**.

Multiple Mass Analysis: 4 mass(es) processed
Tolerance = 5.0 mDa / DBE: min = -1.5, max = 50.0
Element prediction: Off
Number of isotope peaks used for i-FIT = 3

Monoisotopic Mass, Even Electron Ions
927 formula(e) evaluated with 4 results within limits (up to 50 best isotopic matches for each mass)
Elements Used:
C: 0-500 H: 0-1000 O: 0-200
20191216_SeoYH_Gyko7F-J22_pos 328 (3.685) Cm (327:331)
1: TOF MS ES+

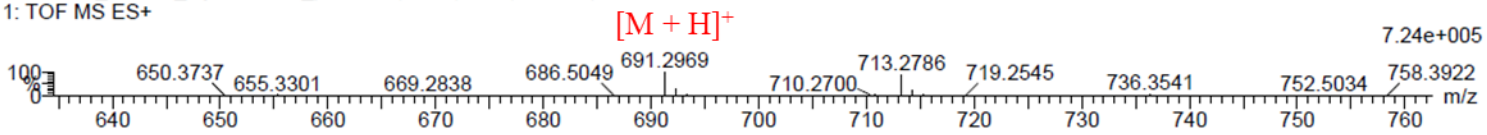

Mass	RA	Calc. Mass	mDa	PPM	DBE	i-FIT	Norm	Conf(%)	Formula
Minimum:	15.00				-1.5				
Maximum:	100.00		5.0	10.0	50.0				
691.2969	100.00	691.2966	0.3	0.4	12.5	102.6	0.024	97.68	C35 H47 O14
		691.3001	-3.2	-4.6	34.5	106.4	3.761	2.32	C53 H39 O
692.3008	28.64	---							
713.2786	85.06	713.2809	-2.3	-3.2	15.5	121.6	0.282	75.42	C37 H45 O14
		713.2751	3.5	4.9	24.5	122.7	1.403	24.58	C44 H41 O9
714.2817	22.36	---							

Figure S9. ^{1}H NMR spectrum (600 MHz, methanol-d_4) of the new compound **2**.

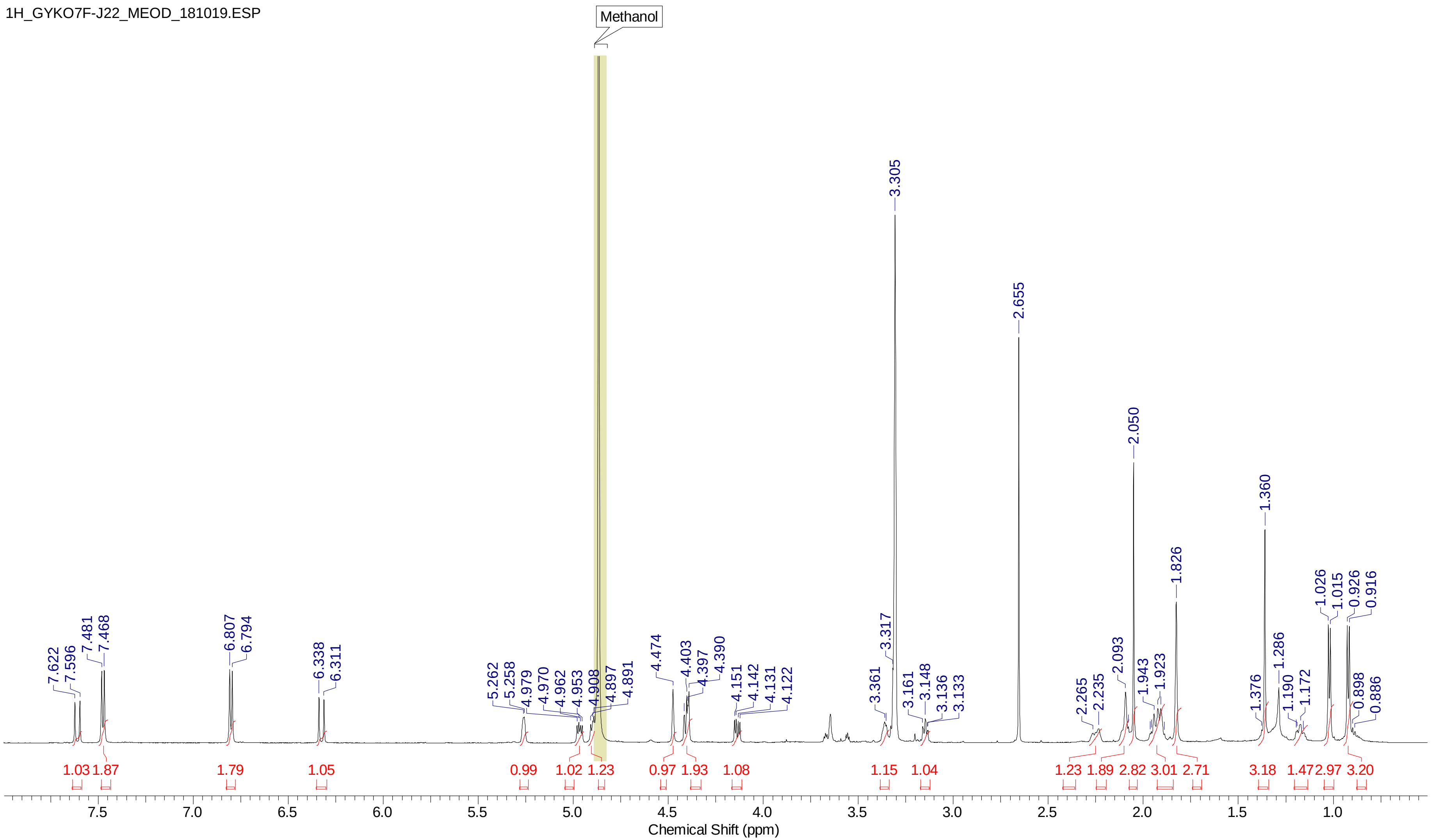

Figure S10. ^{13}C NMR spectrum (150 MHz, methanol-d_4) of the new compound **2**.

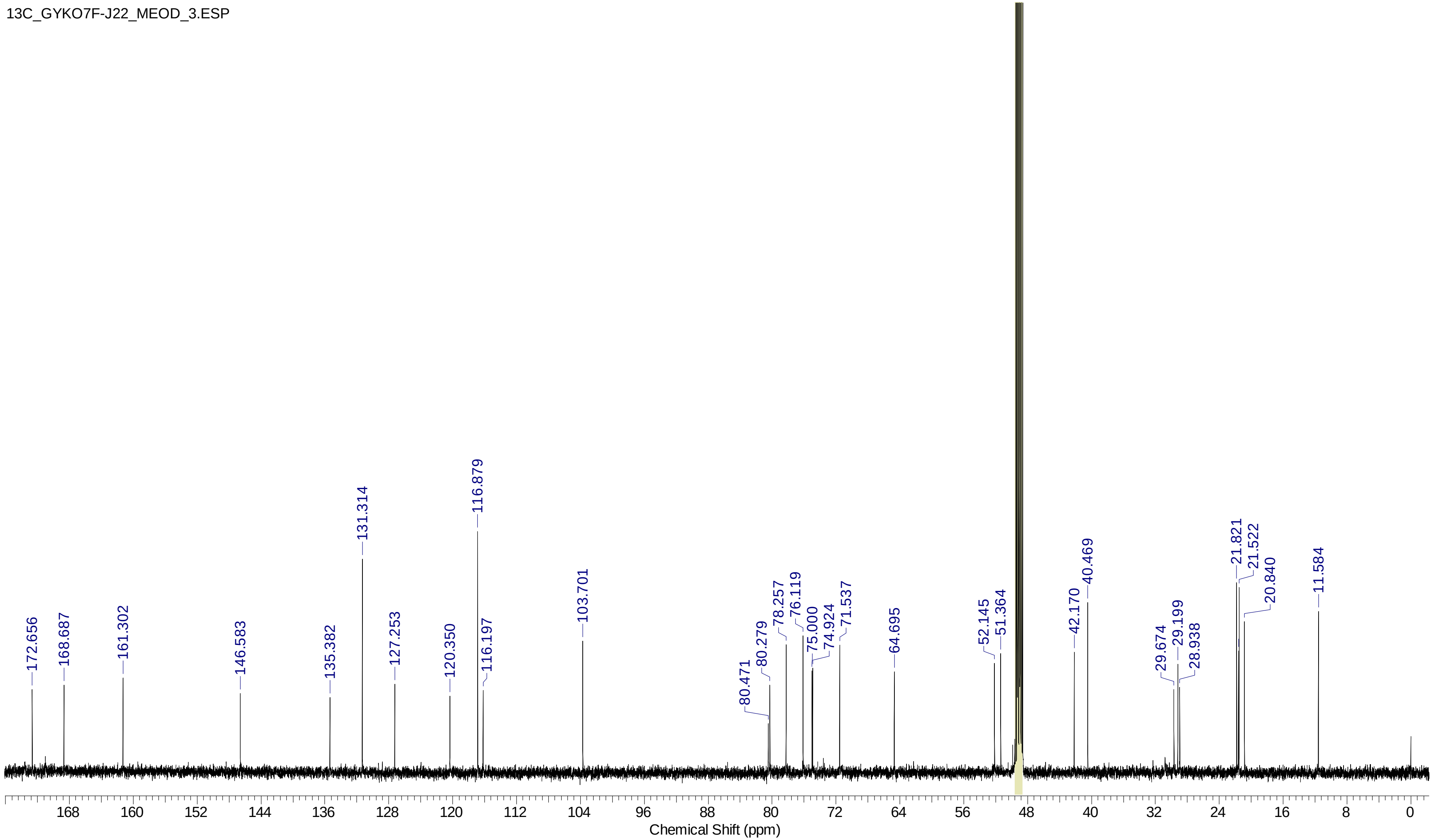

Figure S11. HSQC spectrum (600 MHz, methanol-d_4) of the new compound **2**.

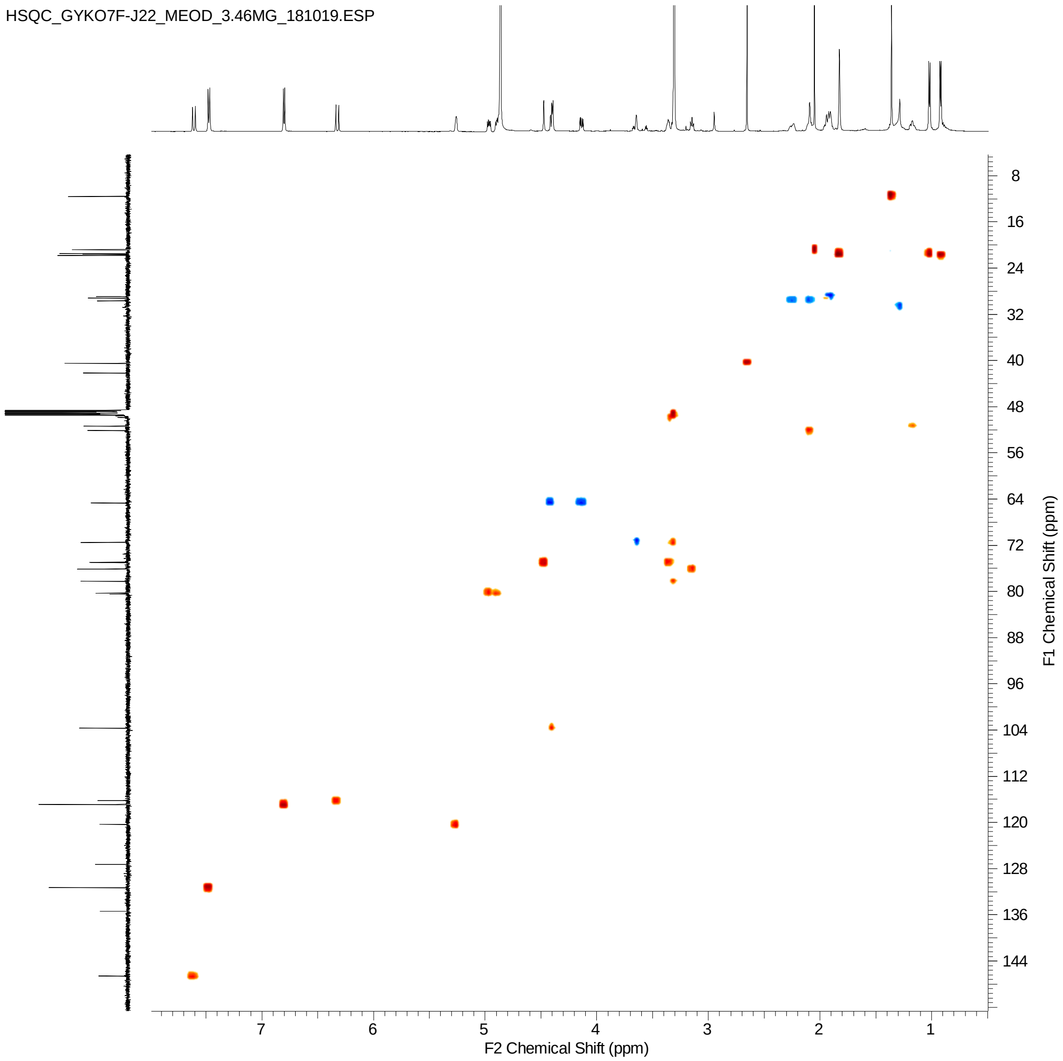

Figure S12. HMBC spectrum (600 MHz, methanol-d_4) of the new compound **2**.

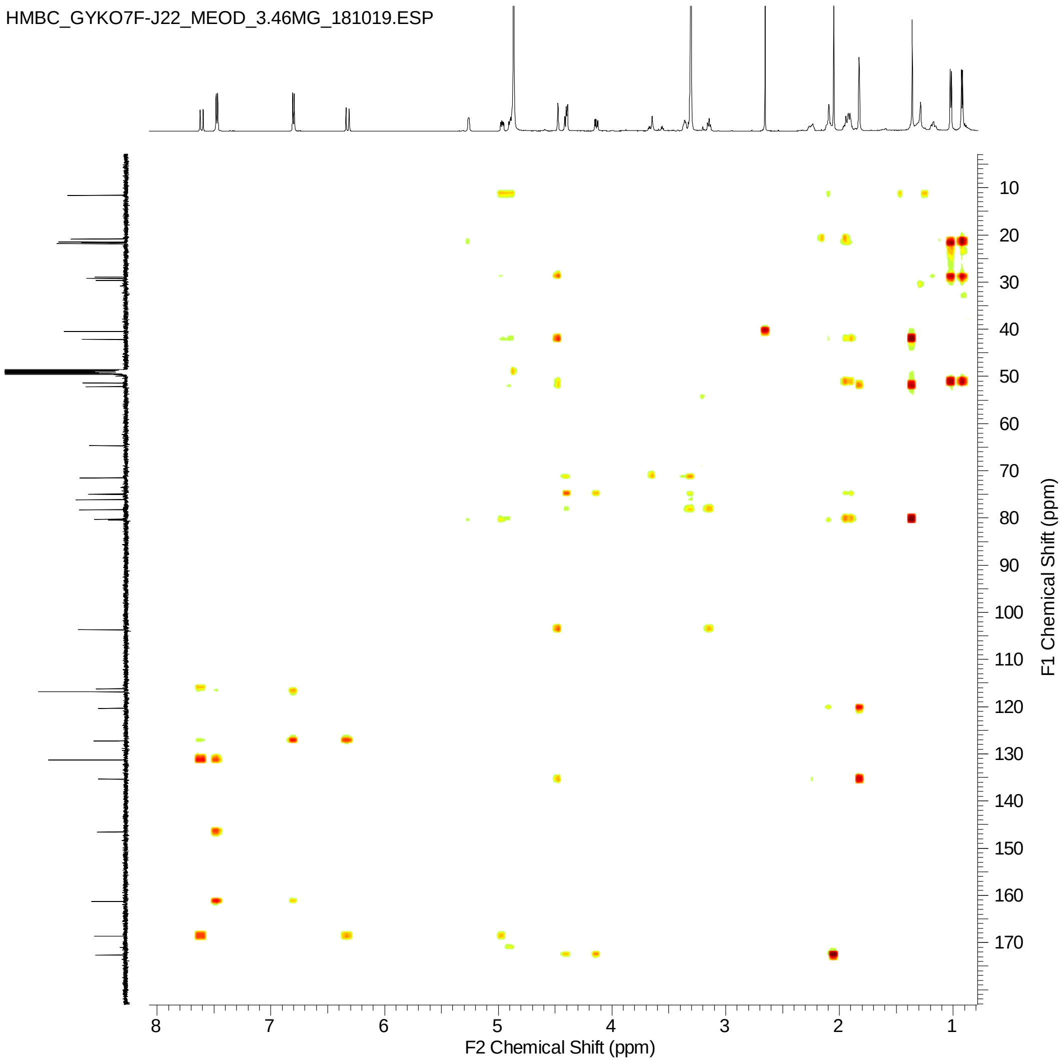

Figure S13. COSY spectrum (600 MHz, methanol-d_4) of the new compound **2**.

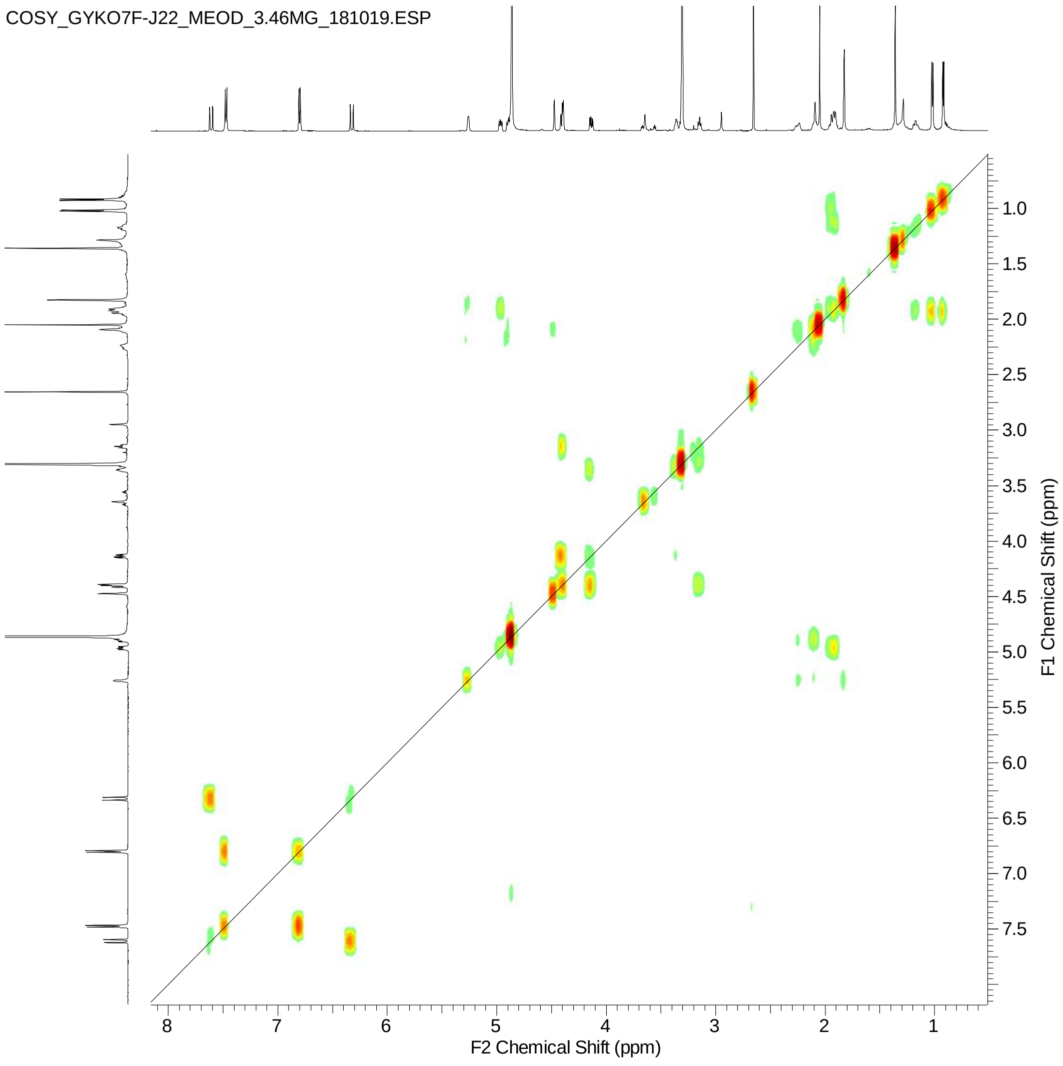

Figure S14. NOESY spectrum (600 MHz, methanol-d_4) of the new compound **2**.

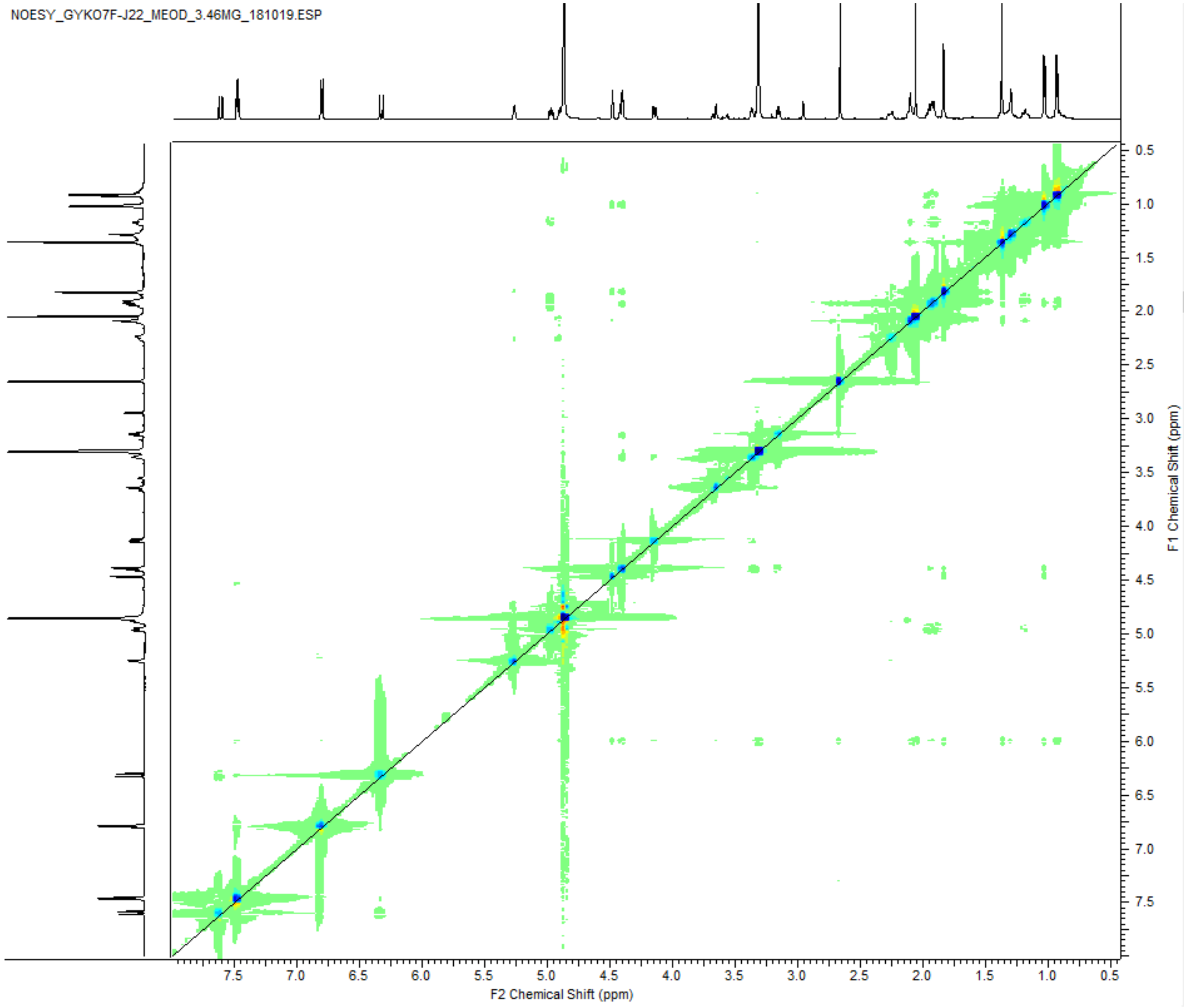

Figure S15. ^{1}H NMR spectrum (700 MHz, DMSO-d_6) of the new compound **2**.

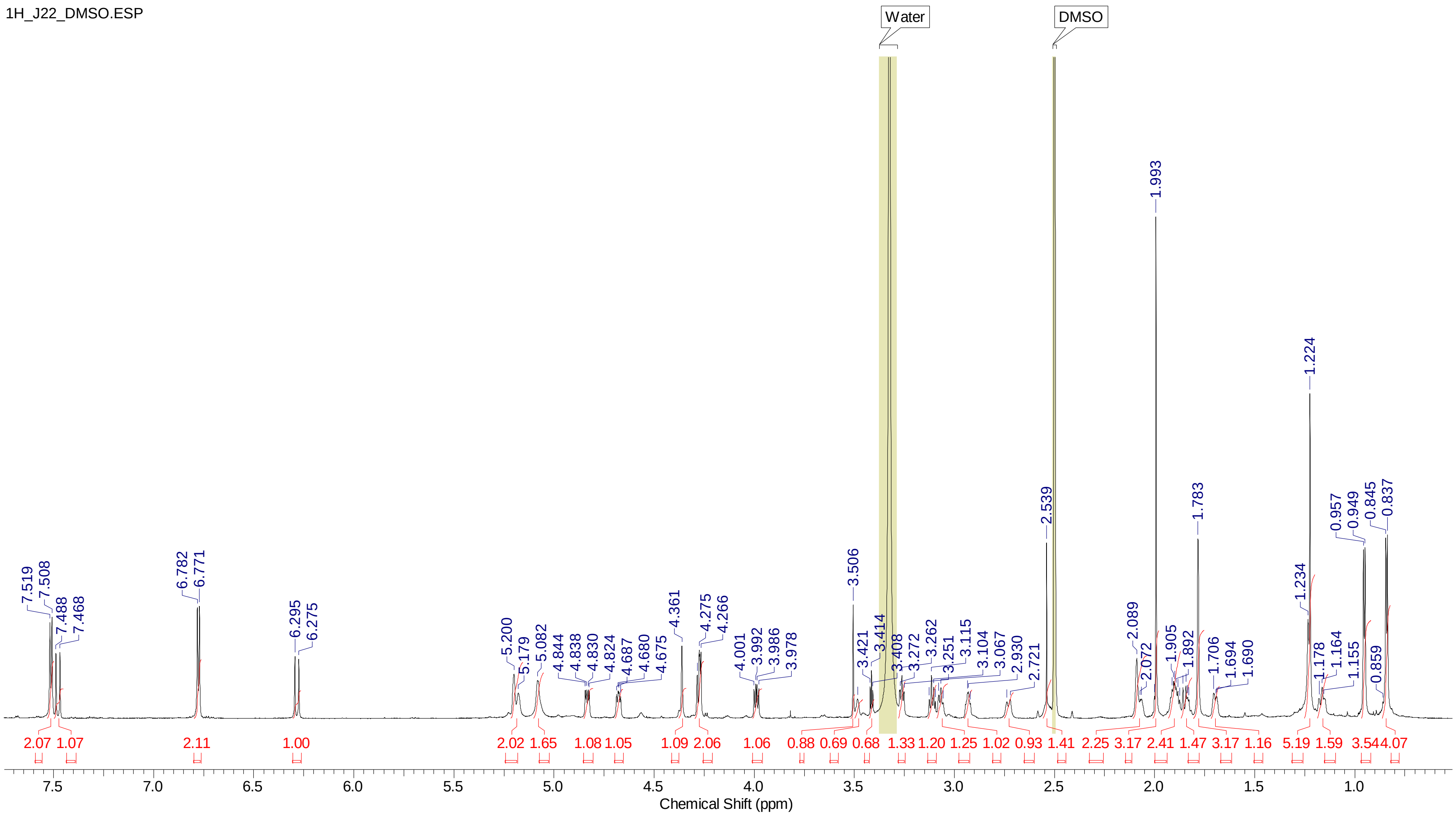

Figure S16. ^{13}C NMR spectrum (175 MHz, DMSO-d_6) of the new compound **2**.

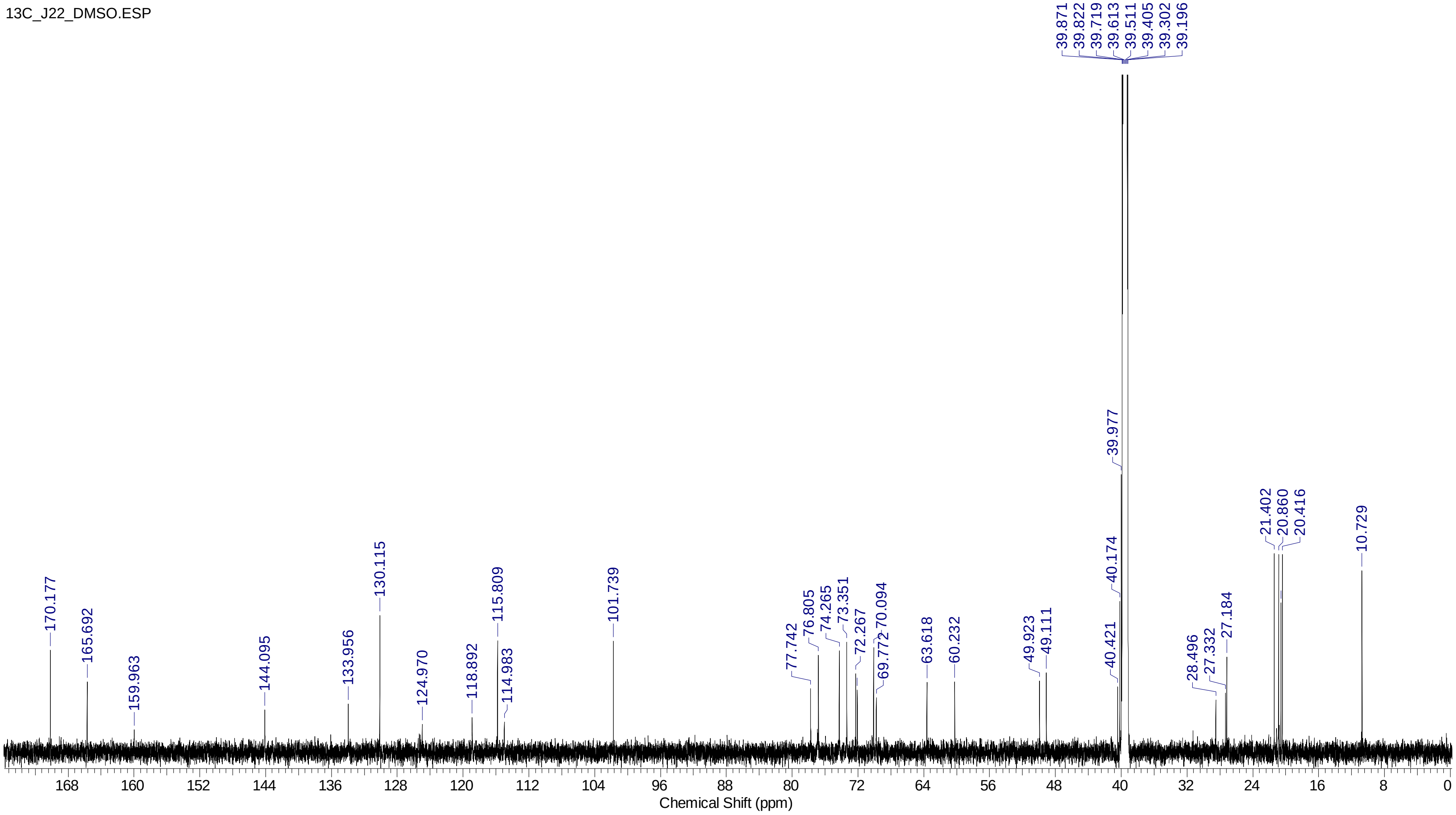

Figure S17. HSQC spectrum (700 MHz, DMSO-d_6) of the new compound **2**.

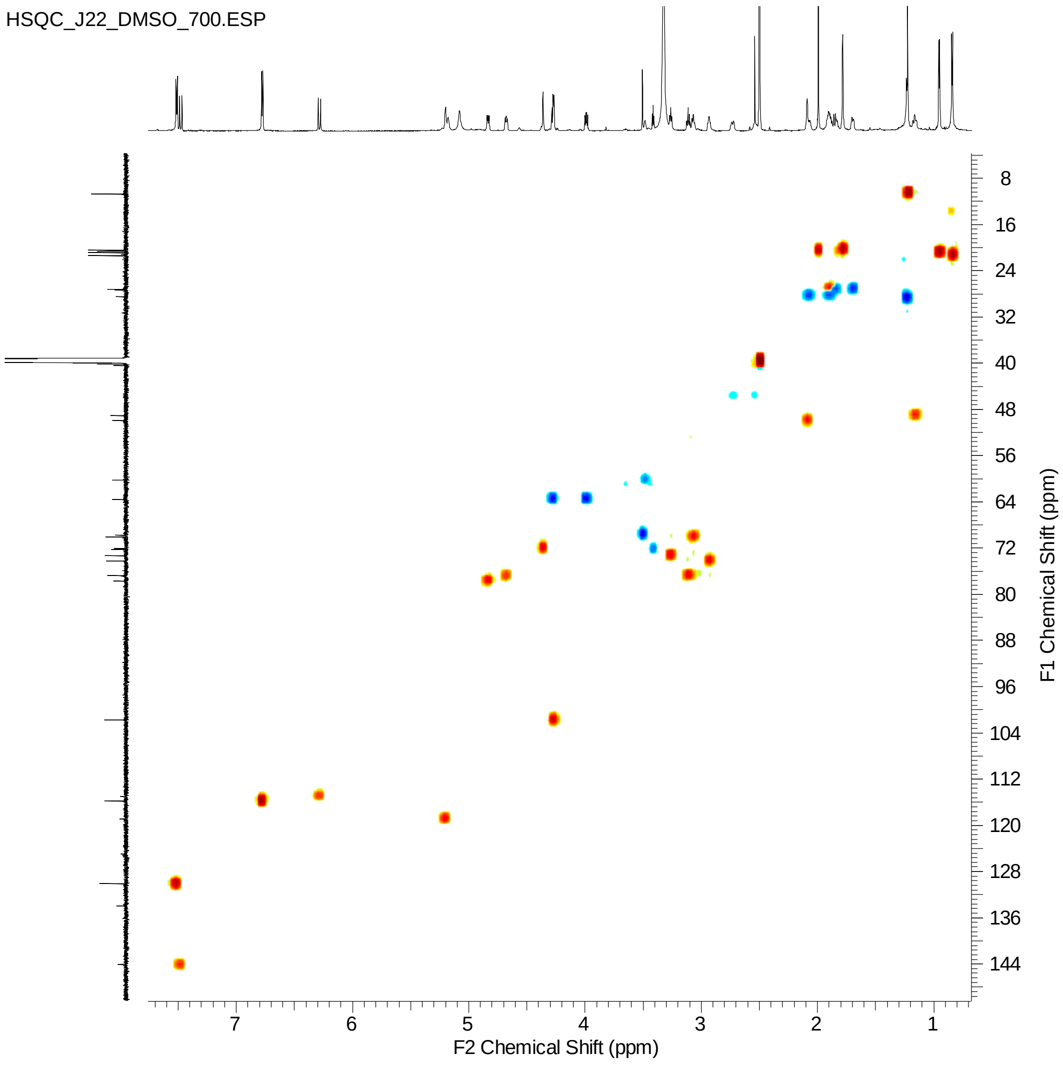

Figure S18. HMBC spectrum (700 MHz, DMSO-d_6) of the new compound **2**.

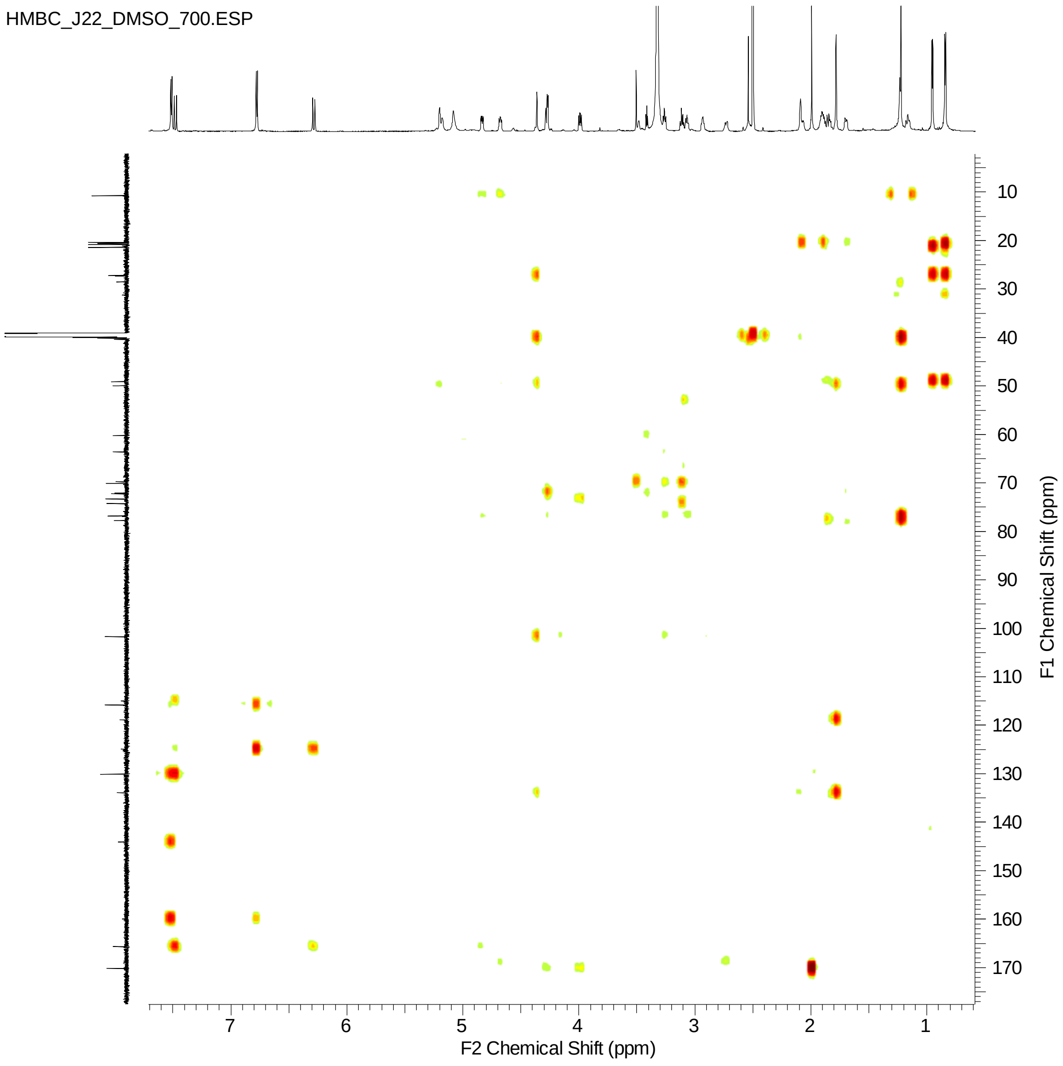

Figure S19. COSY spectrum (700 MHz, DMSO-d_6) of the new compound **2**.

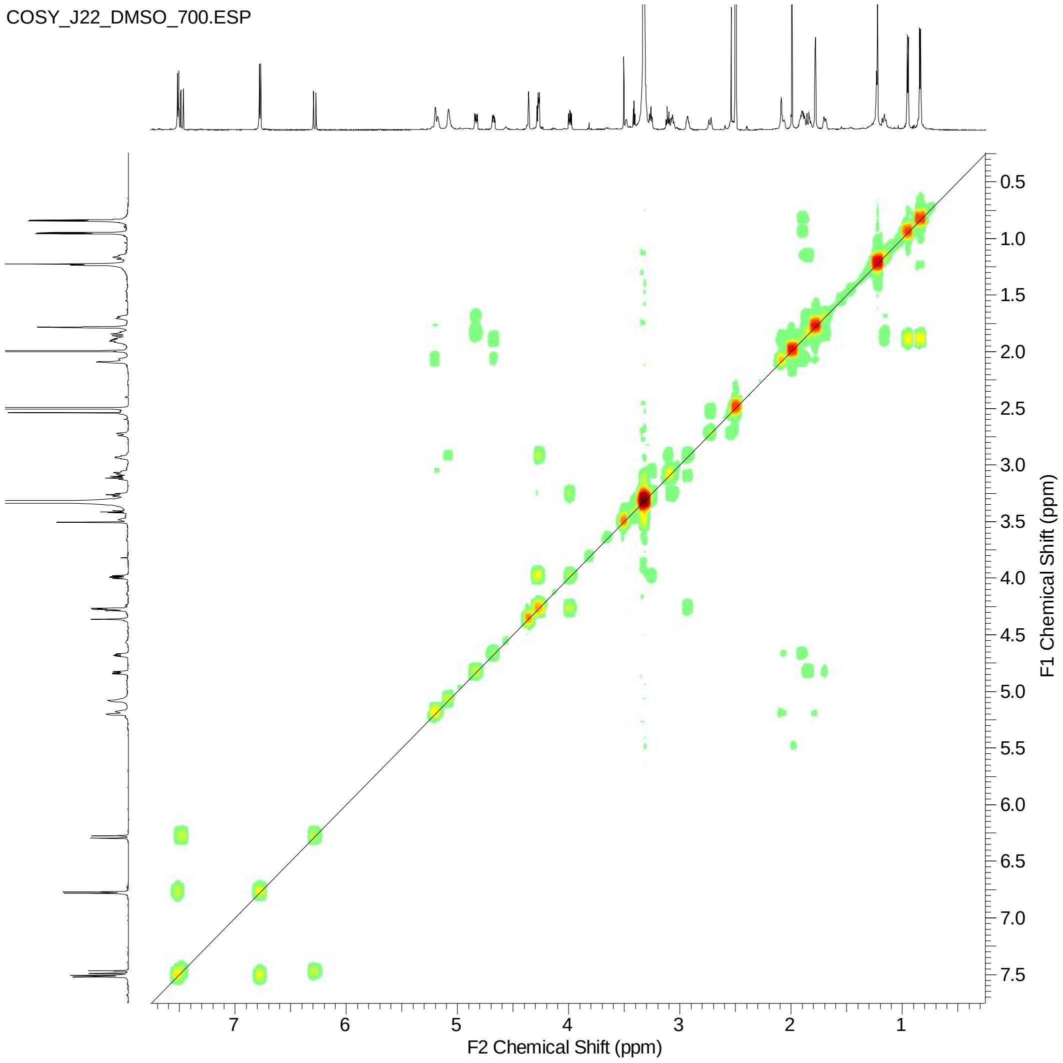

Figure S20. HRESIMS spectrum of the new compound **3**.

Multiple Mass Analysis: 2 mass(es) processed
Tolerance = 5.0 PPM / DBE: min = -1.5, max = 20.0
Element prediction: Off
Number of isotope peaks used for i-FIT = 5

Monoisotopic Mass, Even Electron Ions
385 formula(e) evaluated with 1 results within limits (up to 20 closest results for each mass)
Elements Used:
C: 0-100 H: 0-200 O: 0-50 Na: 0-1
20191216_SeoYH_Gyko7F-J17_pos_re 244 (2.743)
1: TOF MS ES+

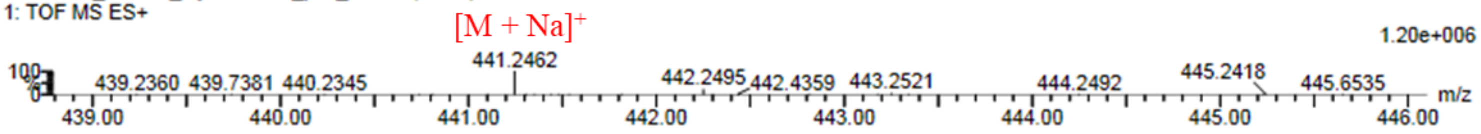

	RA	Calc. Mass	mDa	PPM	DBE	i-FIT	Norm	Conf(%)	Formula
Minimum:	15.00				-1.5				
Maximum:	100.00		5.0	5.0	20.0				

Mass	RA	Calc. Mass	mDa	PPM	DBE	i-FIT	Norm	Conf(%)	Formula
441.2462	100.00	441.2464	-0.2	-0.5	2.5	145.5	n/a	n/a	C21 H38 O8 Na

Figure S21. ^{1}H NMR spectrum (600 MHz, methanol-d_4) of the new compound **3**.

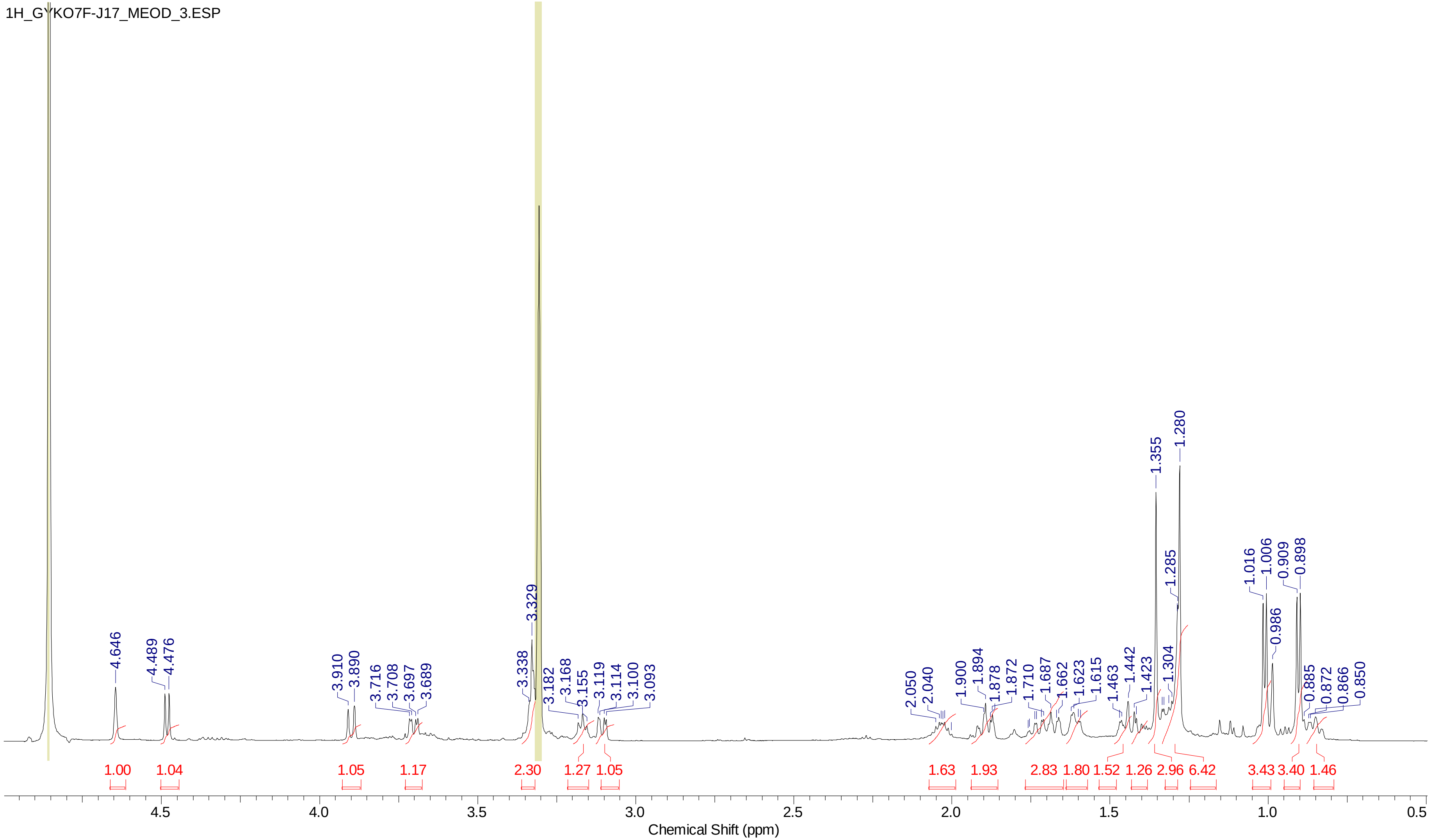

Figure S22. ^{13}C NMR spectrum (150 MHz, methanol-d_4) of the new compound **3**.

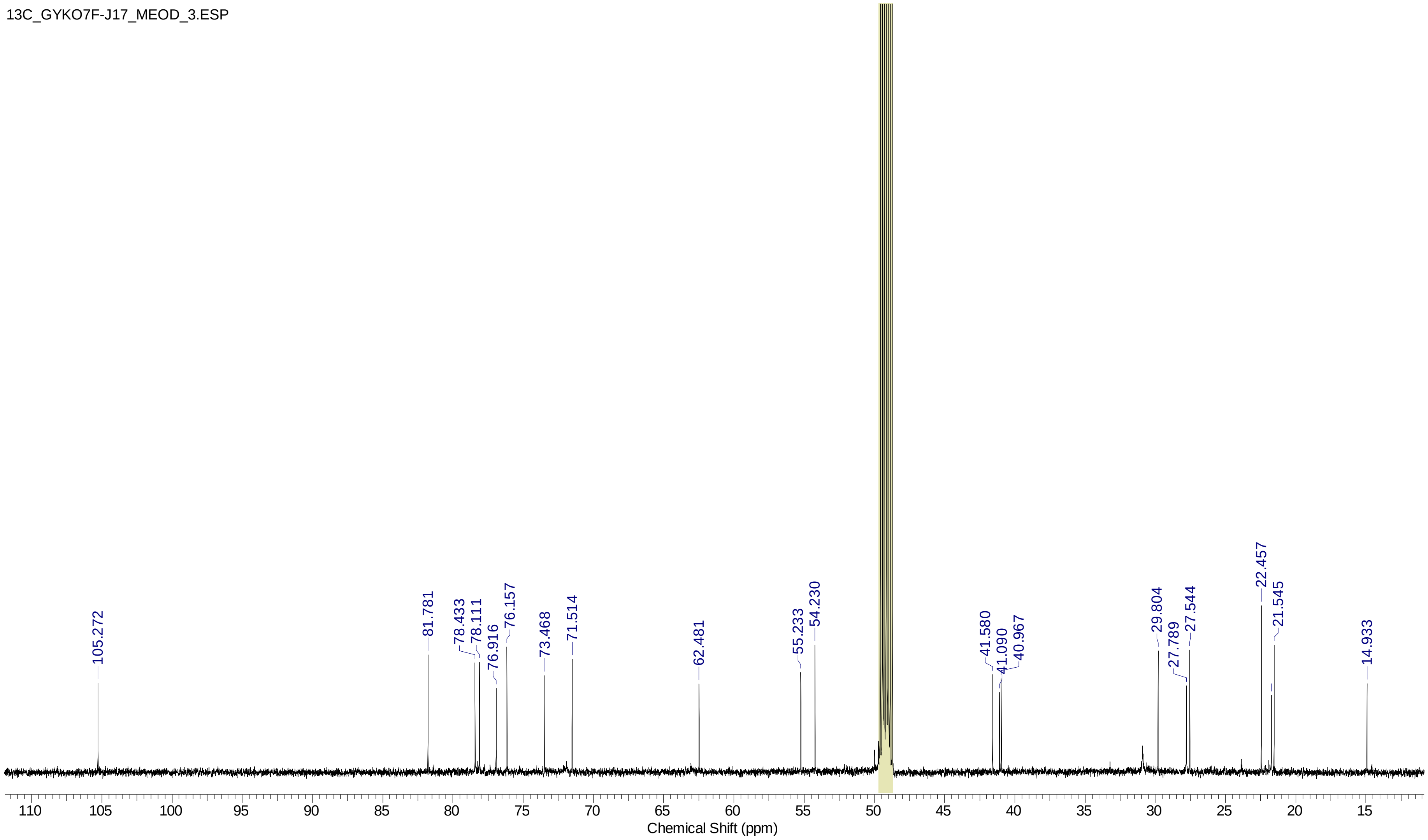

Figure S23. HSQC spectrum (600 MHz, methanol-d_4) of the new compound **3**.

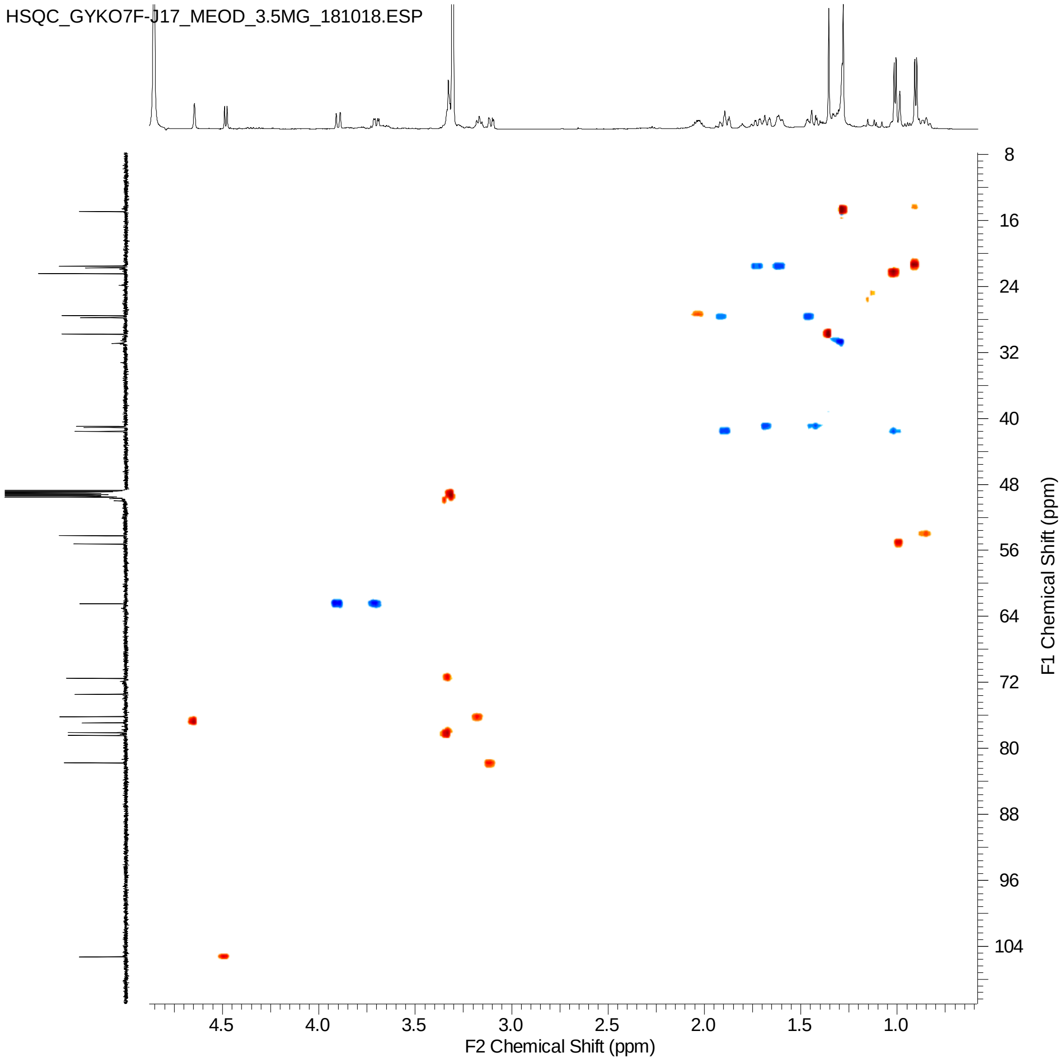

Figure S24. HMBC spectrum (600 MHz, methanol-d_4) of the new compound **3**.

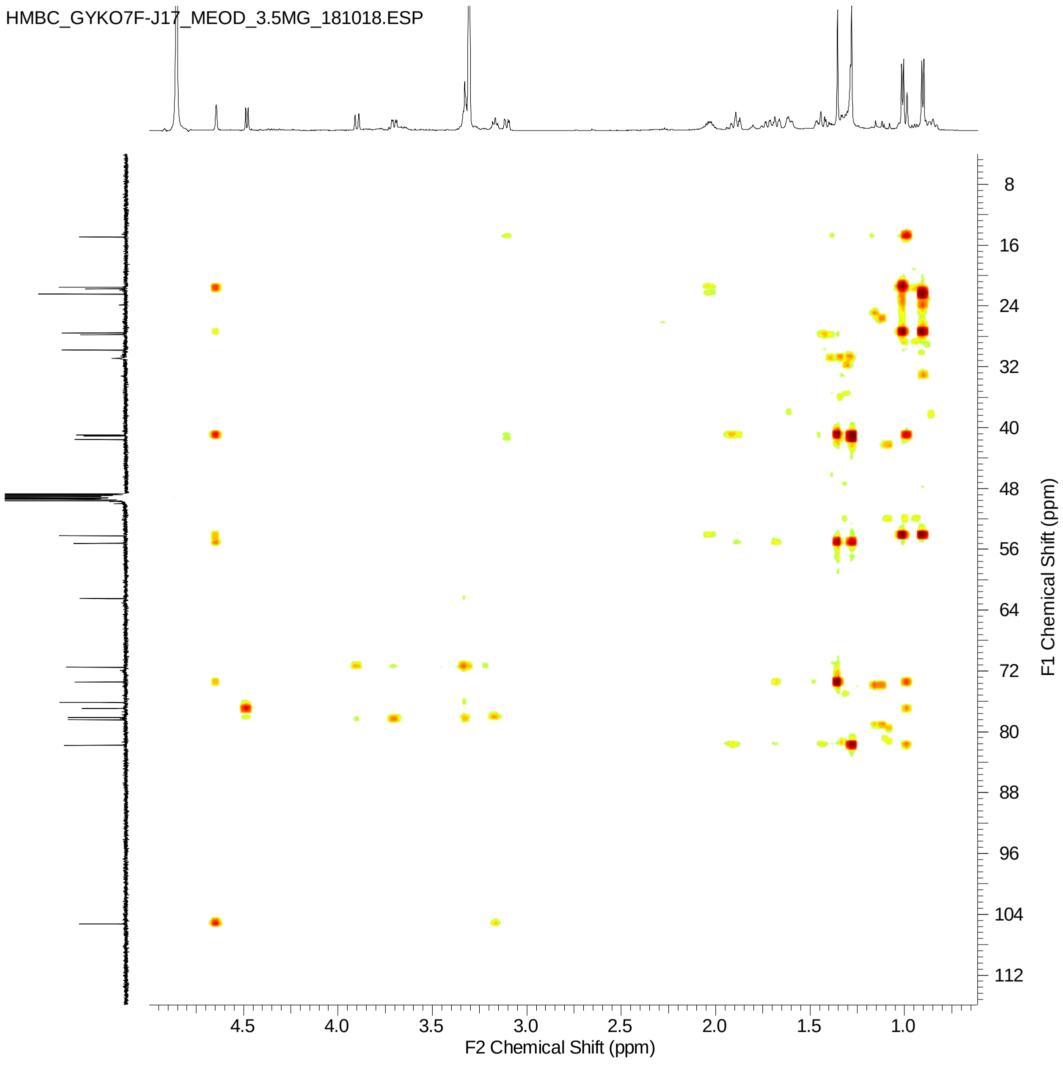

Figure S25. COSY spectrum (600 MHz, methanol-d_4) of the new compound **3**.

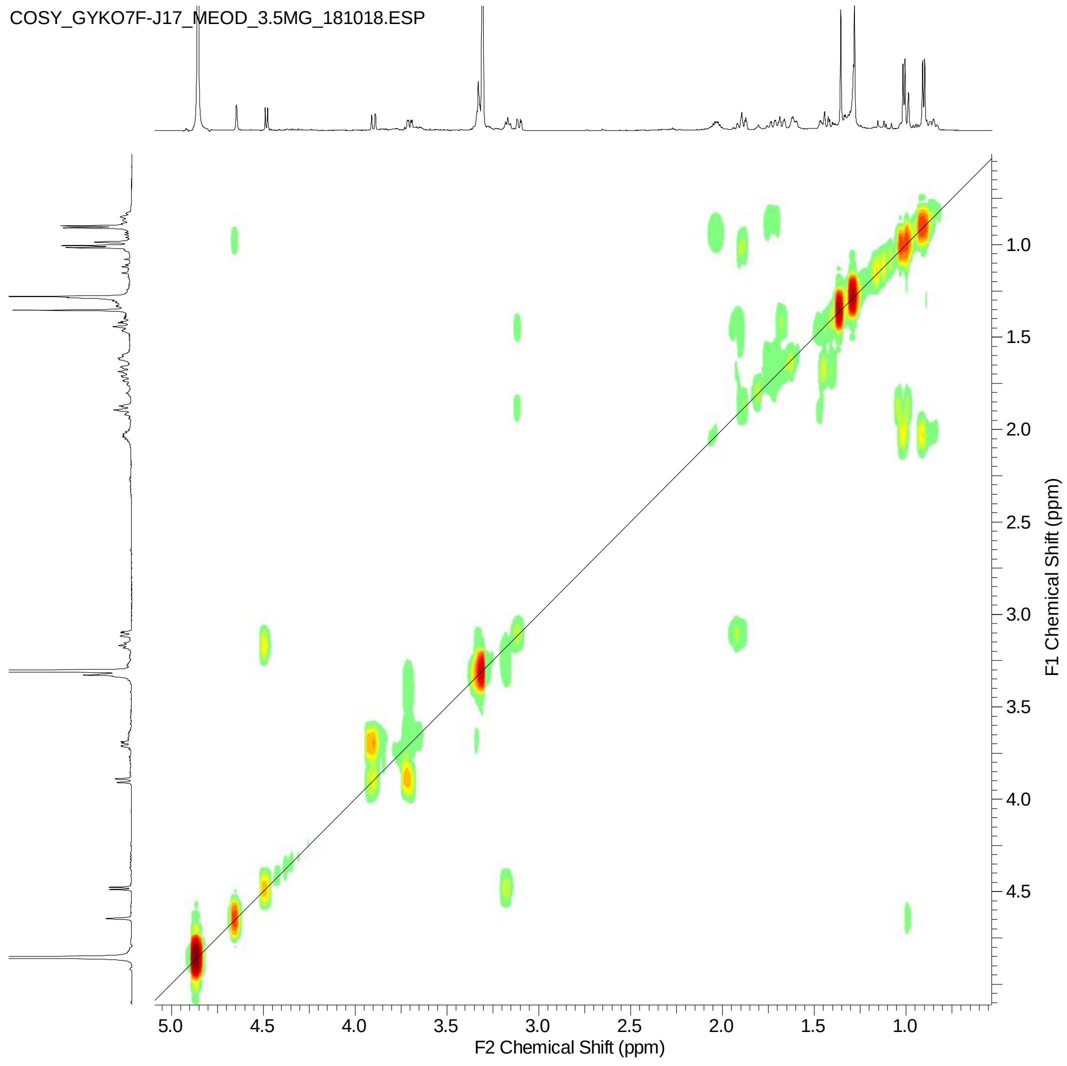

Figure S26. NOESY spectrum (600 MHz, methanol-d_4) of the new compound **3**.

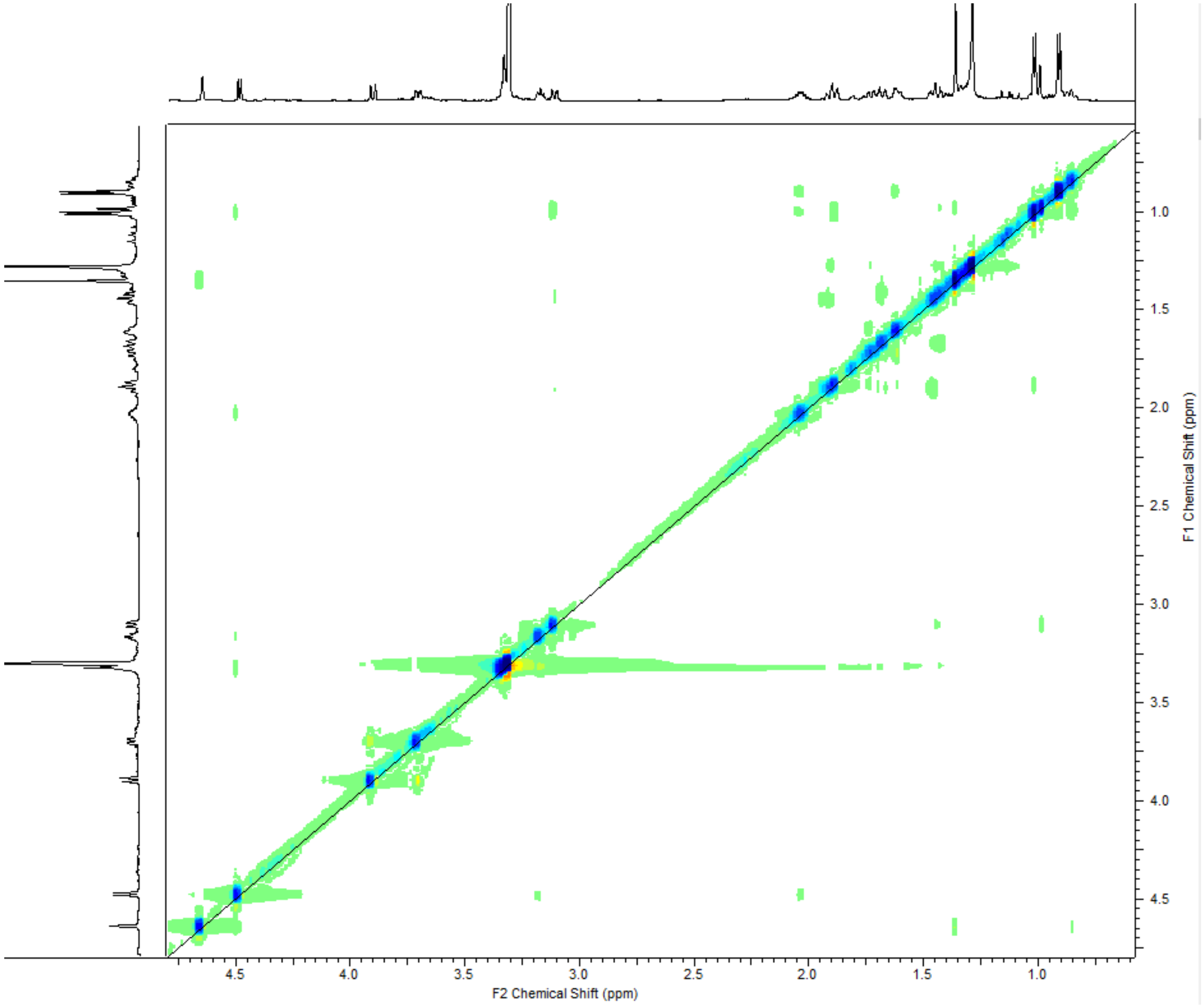

Figure S27. HRESIMS spectrum of the new compound **4**.

Multiple Mass Analysis: 2 mass(es) processed
Tolerance = 5.0 PPM / DBE: min = -1.5, max = 20.0
Element prediction: Off
Number of isotope peaks used for i-FIT = 5

Monoisotopic Mass, Even Electron Ions
652 formula(e) evaluated with 2 results within limits (up to 20 closest results for each mass)
Elements Used:
C: 0-100 H: 0-200 O: 0-50 Na: 0-1
20191216_SeoYH_Gyko7F-J19_pos_re 231 (2.600)
1: TOF MS ES+

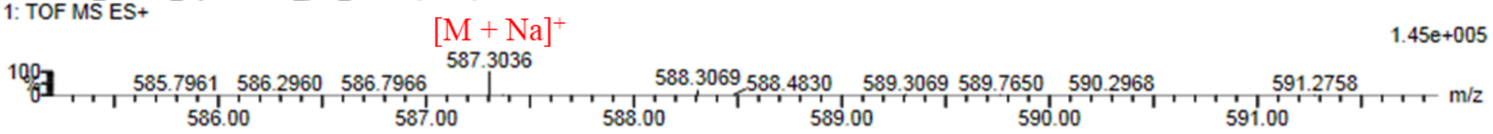

Minimum:	15.00				-1.5				
Maximum:	100.00		5.0	5.0	20.0				
Mass	RA	Calc. Mass	mDa	PPM	DBE	i-FIT	Norm	Conf(%)	Formula
587.3036	100.00	587.3009	2.7	4.6	15.5	63.1	1.273	28.00	C36 H43 O7
		587.3043	-0.7	-1.2	3.5	62.1	0.328	72.00	C27 H48 O12 Na

Figure S28. ^{1}H NMR spectrum (600 MHz, methanol-d_4) of the new compound **4**.

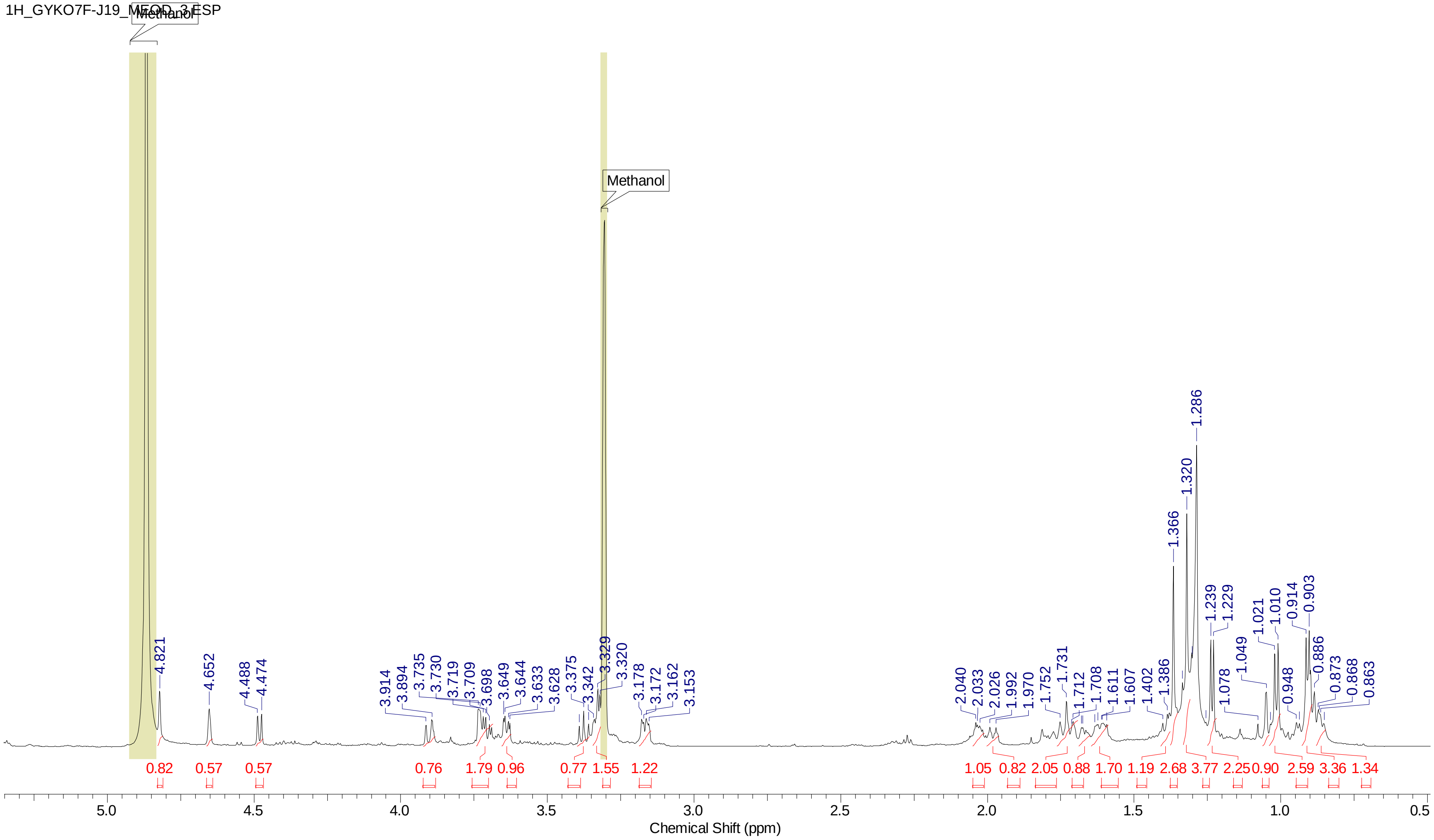

Figure S29. ^{13}C NMR spectrum (150 MHz, methanol-d_4) of the new compound **4**.

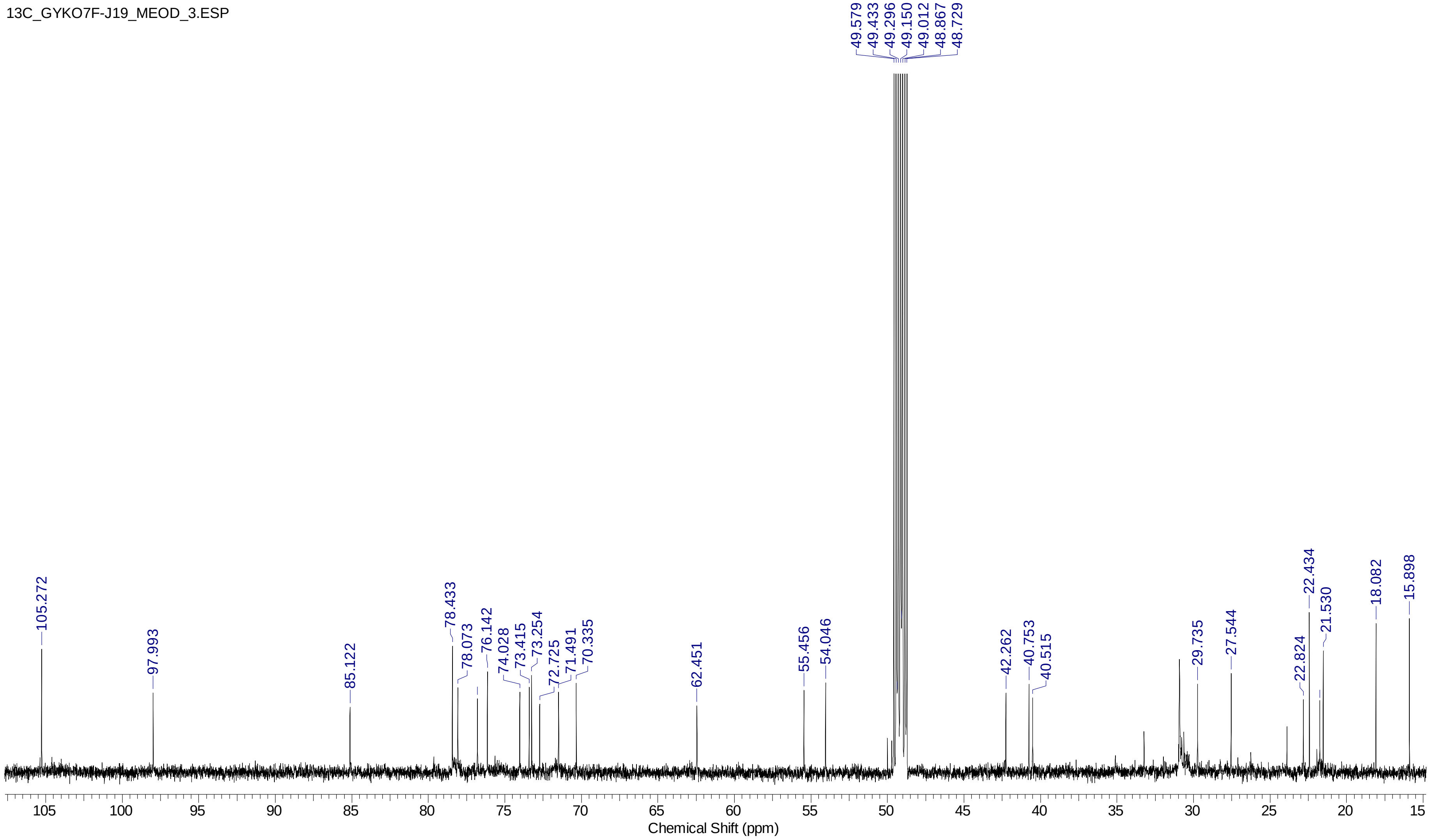

Figure S30. HSQC spectrum (600 MHz, methanol-d_4) of the new compound **4**.

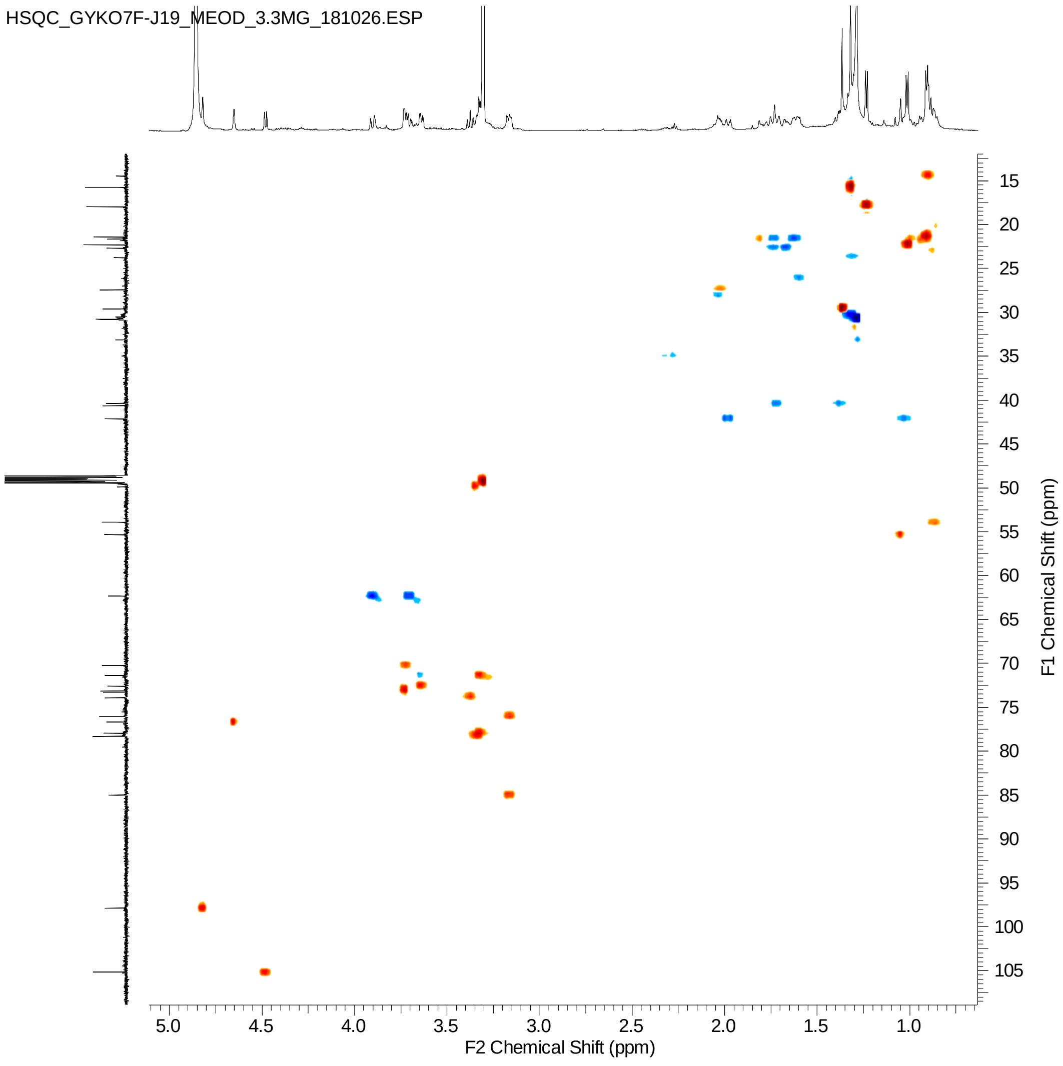

Figure S31. HMBC spectrum (600 MHz, methanol-d_4) of the new compound **4**.

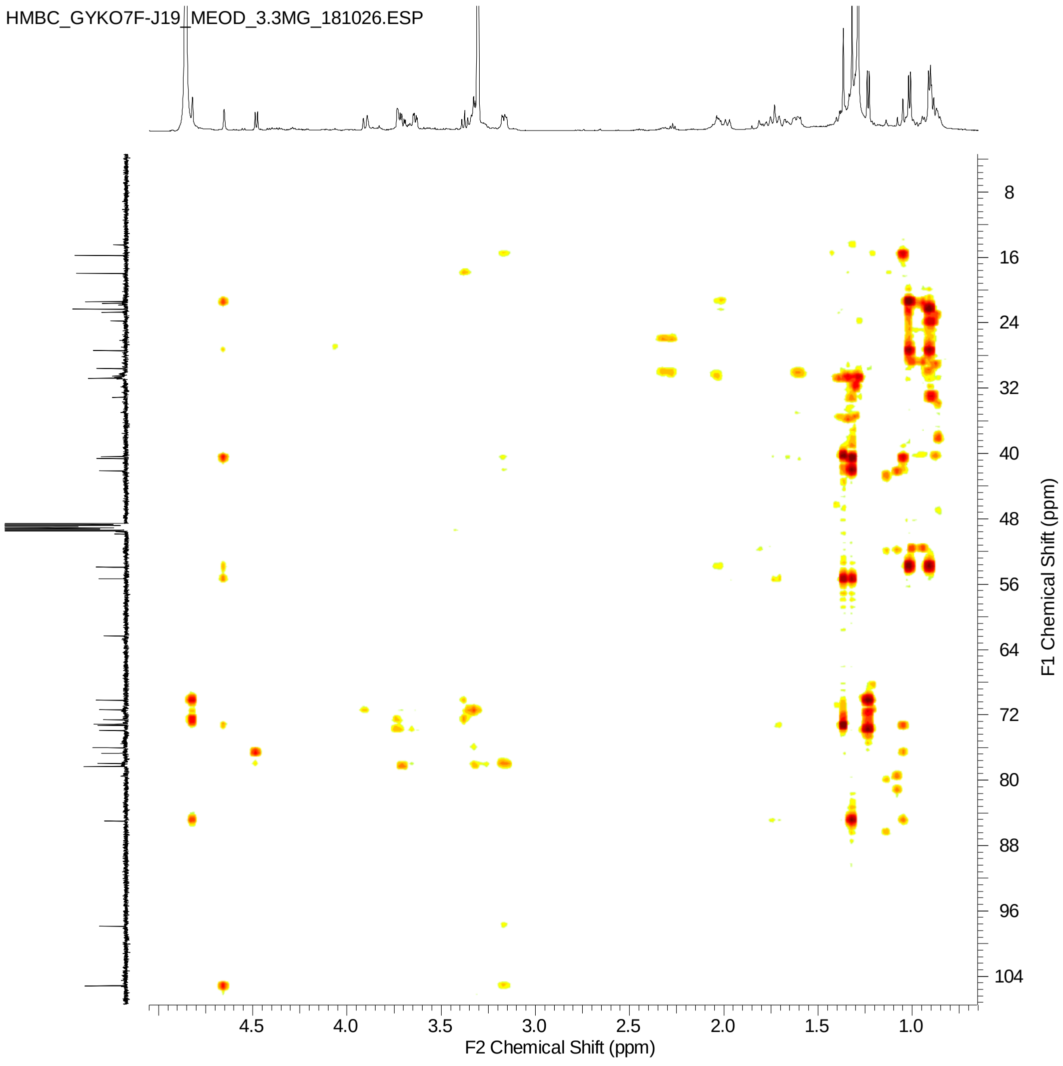

Figure S32. COSY spectrum (600 MHz, methanol-d_4) of the new compound **4**.

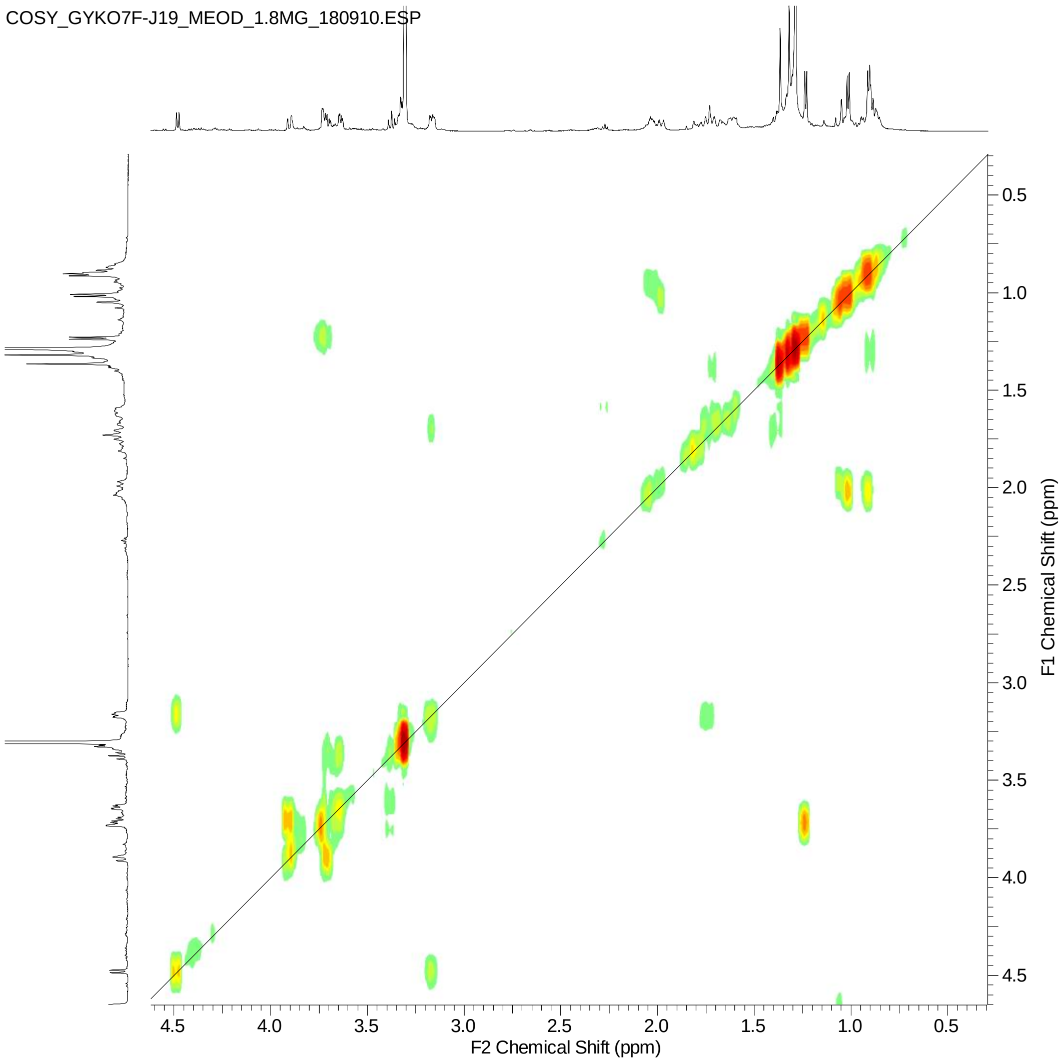

Figure S33. NOESY spectrum (600 MHz, methanol-d_4) of the new compound **4**.

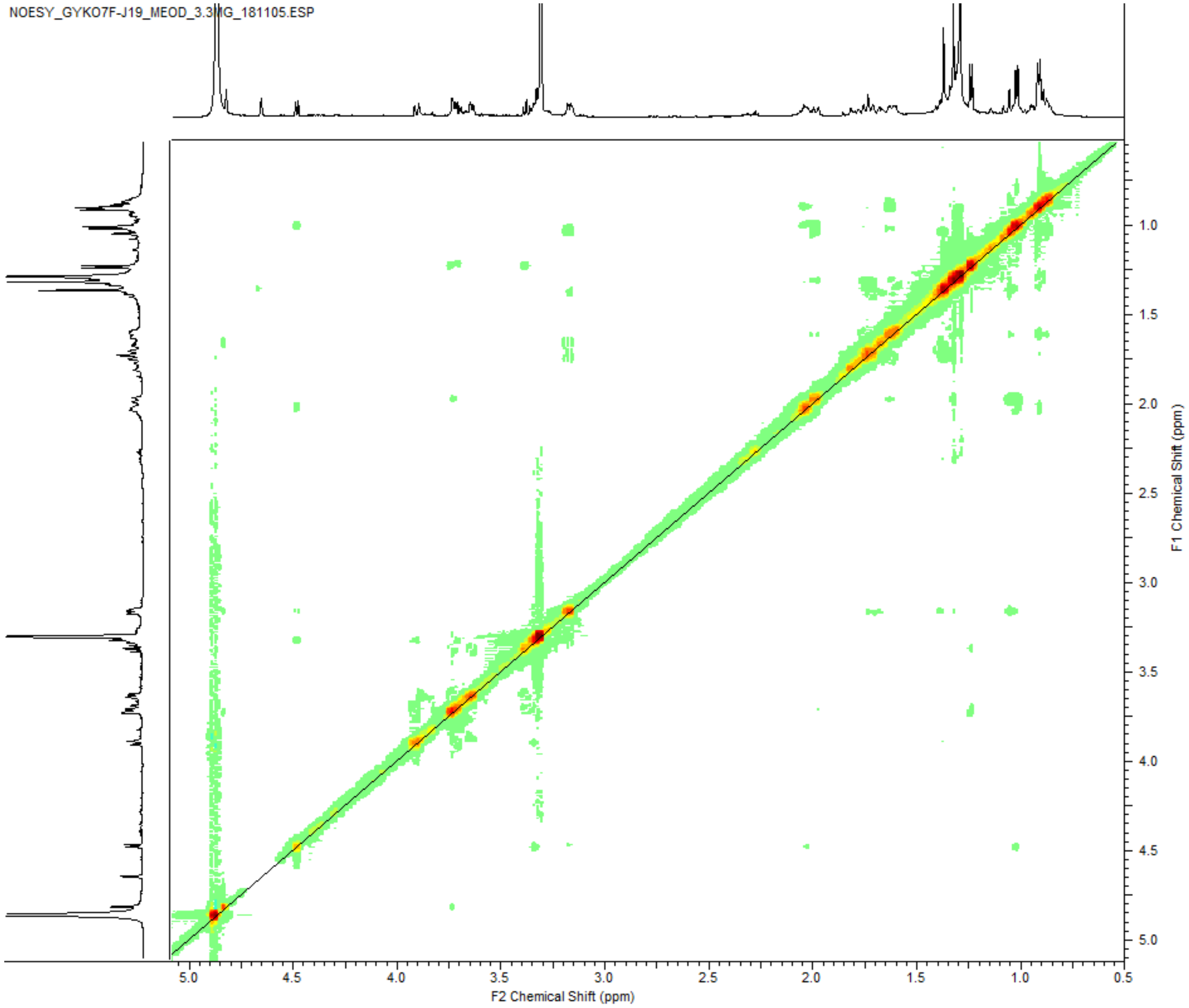

Supplement: Supplementary file 1 [file plants-12-01726-s001.zip › plants-2203177-supplementary.pdf]
